# Fstl1 determines luminal size of mouse trachea through negative regulation of BMP4 signaling

**DOI:** 10.1016/j.isci.2026.116972

**Published:** 2026-07-30

**Authors:** Yueyue Jin, Yue Zhang, Yingying Liu, Maolin Yao, Chenjie Zhang, Shuzi Li, Yibo Ding, Zhuan Niu, Yue Han, Yan Geng, Lian Li, Xue Liu, Wen Ning

**Affiliations:** 1 State Key Laboratory of Medicinal Chemical Biology, College of Life Sciences, Nankai University, Tianjin 300071, China; 2 School of Pharmaceutical Science, Jiangnan University, Wuxi 214122, Jiangsu, China; 3 Department of Medicine and Women’s Guild Lung Institute, Cedars-Sinai Medical Center, Los Angeles, CA 90048, USA

**Keywords:** tracheal morphogenesis, luminal epithelial morphogenesis, spindle angle distribution, Follistatin-like 1, Fstl1, BMP4 signaling

## Abstract

Trachea is a wide tube with smooth luminal epithelia that enables efficient ventilation. In this study, we provide the evidence that the mitotic angles of most tracheal epithelial cells exhibit ordered and transverse orientations, which allows the tracheal lumen to grow along the anterior-posterior (AP) axis, with its circumference being higher than length. We show that the BMP4 signaling antagonist follistatin-like 1 (Fstl1) is a critical regulator for tracheal morphogenesis. Mice with *Fstl1* deletion exhibit wider trachea with enlarged lumen as a result of the increased proportion of mitotic cells with their spindles nearly vertical to the tracheal longitudinal axis. Mechanistically, Fstl1 regulates BMP4 signaling by antagonizing *p*-Smad1/5/8 in tracheal epithelial cells. Reducing BMP4 signaling activity rescues lumen enlargement and normalizes the spindle angle distribution in *Fstl1*-deficient tracheas. Therefore, we provide evidence to demonstrate that Fstl1 modulates the oriented cell division and tracheal morphogenesis, in part, through BMP4 signaling.

## Highlights

• Fstl1 deficiency increases embryonic mouse tracheal lumen
• Fstl1 regulates mesenchymal-epithelial crosstalk via Bmp4/Smad pathway
• Aberrant BMP4 signaling causes abnormal tracheal epithelial mitotic spindle angles
• Blocking BMP4 signaling rescues enlarged tracheal lumen in Fstl1-deficient trachea

## Graphical abstract

**Figure:**
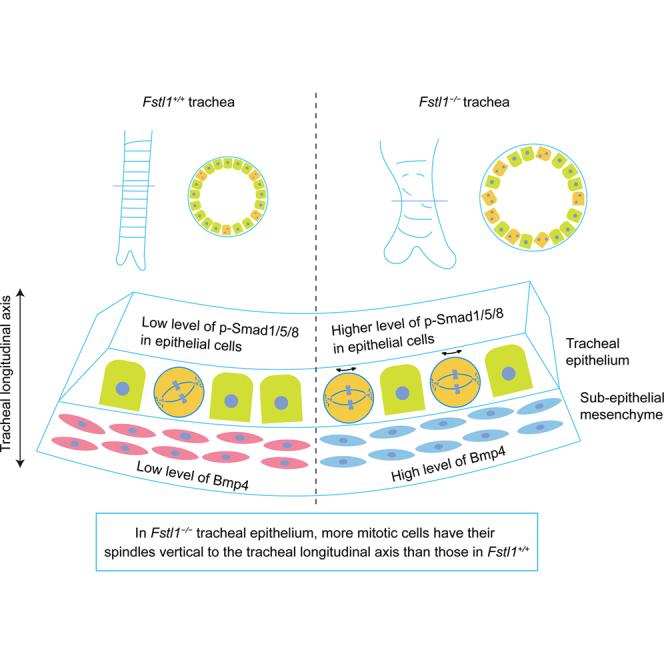

## Teaser

Molecular biology; Molecular interaction; Cell biology

## Introduction

The trachea is an exclusive passage that connects the larynx to the bronchi to deliver external air to the lungs. To ensure ventilation efficiency, the trachea develops a wide tubular morphology with smooth luminal epithelium, surrounded by C-shaped cartilage rings and smooth muscle stripe (SM), which balance the rigidity and elasticity of the airway to prevent collapse.^1^^,^^2^ In mice, the tracheal morphology occurs following lineage specification (embryonic day 8.5 [E8.5]–E10.5) and undergoes sequential events of tracheoesophageal separation (E9.5–E12.5) and tube elongation/expansion (E10.5–E18.5).^3^ Tracheal morphology is determined during the elongation/expansion stage, which is initiated and regulated by the mesenchymal tissues, including the cartilage and SM.^3^^,^^4^^,^^5^^,^^6^ Anomalies in their formation can result in life-threatening malformation of the trachea, such as tracheomalacia and tracheostenosis.^7^^,^^8^

As the trachea grows in both length and width, its luminal epithelial surface enlarges whilst developing into pseudostratified epithelial layers at the lumen. The cellular basis determining the lumen size of developing mouse trachea has not yet been elucidated. Studies have revealed that the increase in lumen size mainly relies on cell proliferation during early development until E14.5 but on expansion of the cell apical surface and modest cell proliferation after E14.5.^3^^,^^4^ In the lungs, oriented division of proliferating cells by the RAS-ERK1/2 signaling has been reported to determine the lumen size of airway tubes.^9^ Tang et al.^9^ measured the mitotic spindle angle of airway epithelial cells and showed that longitudinal orientation allows the tube length to increase more than circumference. Although both the trachea and the lung airways elongate along the anterior-posterior (AP) axis in early stages, their lumen morphogenesis is quite different. The potential role of oriented cell division on tracheal lumen size determination and mechanisms underlying remain largely unknown.

Follistatin-like 1 (Fstl1) is a small glycoprotein primarily synthesized by mesenchymal-derived cells and belongs to the SPARC (secreted protein acidic and rich in cysteine) family of matricellular proteins.^10^ Although functions and mechanisms of Fstl1 are not completely understood, a rapidly expanding body of literature has described the involvement of Fstl1 in embryonic development, tissue remodeling/repair, and inflammatory processes and has identified the regulatory role of Fstl1 in cell growth, differentiation, apoptosis, and migration. We have previously generated *Fstl1*^−/−^ mice and observed the postnatal lethality as a result of respiratory failure. Analyses of the mutant phenotypes have revealed the important role of Fstl1 in the differentiation/maturation of alveolar epithelial cells and demonstrated that Fstl1 is a bone morphogenetic protein (BMP) 4 signaling antagonist that plays a role in controlling mouse lung development.^11^ Meanwhile, we have also observed malformed tracheal tubes with interrupted cartilages, narrowed SM, as well as enlarged lumen size in *Fstl1*^−/−^ mice (E18.5).^11^^,^^12^

In this study, we measured the mitotic spindle angles of the dividing cells and found that most tracheal epithelial cells are transversely oriented with their spindles nearly vertical to the tracheal longitudinal axis. We report that the deletion of *Fstl1* in mice leads to lumen enlargement due to an increased proportion of mitotic epithelial cells with transverse orientation. Reducing BMP4 signaling activity by dorsomorphin or Noggin normalized spindle angle distribution and rescued lumen enlargement in *Fstl1*^−/−^ tracheas. Our study highlights that the Fstl1-downregulated Bmp4 axis plays an important role in the maintenance of the biased spindle angle distribution for lumen size determination of the developing mouse trachea.

## Results

### Lumen enlargement in *Fstl1*^−/−^ tracheas

**Figure 1:**
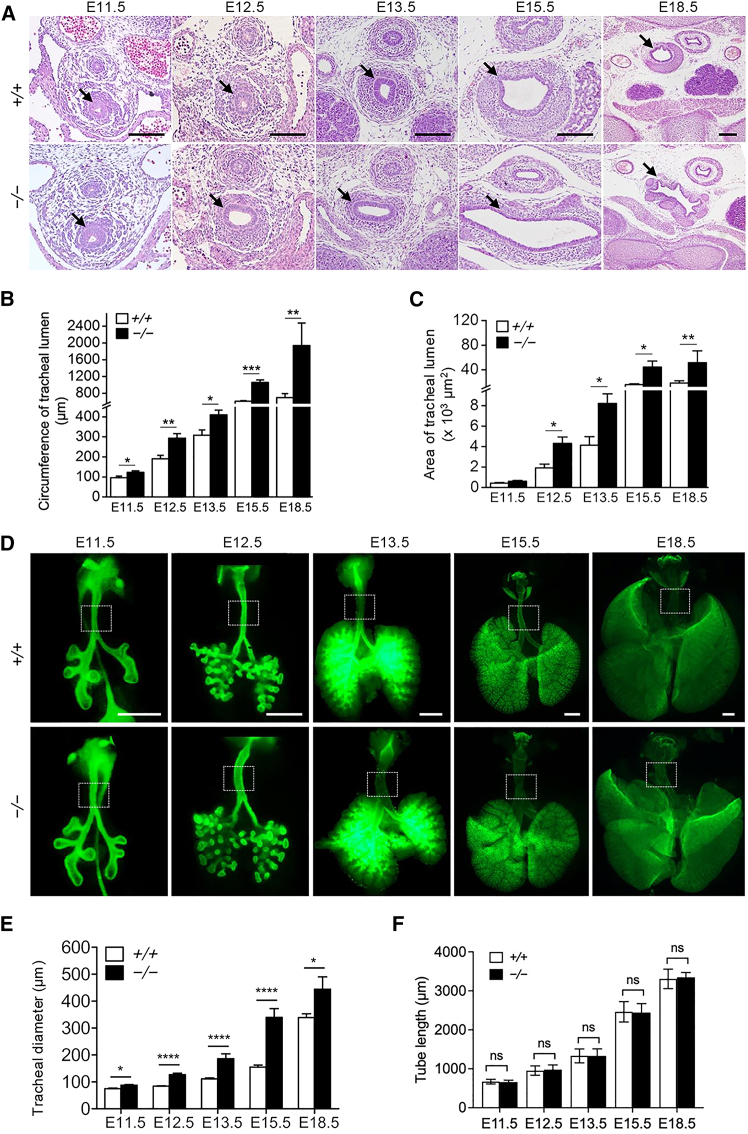
Enlarged tracheal lumen in *Fstl1*-deficient embryos (A) Representative images of H&E staining on tracheal transverse sections from *Fstl1*^*+/+*^ and *Fstl1*^−/−^ mouse embryos at different developmental stages. Scale bars: 100 μm. (B and C) Tracheal lumen circumference (B) and area (C) of embryos at different developmental stages were quantified by bar plots. E11.5: *Fstl1*^*+/+*^, *n* = 11, *Fstl1*^−/−^, *n* = 8; E12.5: *n* = 5; E13.5: *n* = 3; E15.5: *n* = 5; E18.5: *Fstl1*^*+/+*^, *n* = 4, *Fstl1*^−/−^, *n* = 8. ∗, *p* < 0.05, ∗∗, *p* < 0.01, and ∗∗∗, *p* < 0.001*.* (D) Representative images of whole-mount staining of embryonic tracheas and lungs for the epithelial marker E-cadherin to visualize the tracheal lumens. Scale bars: 500 μm. (E) Quantification of tracheal lumen diameters after whole-mount staining. E11.5: *Fstl1*^*+/+*^, *n* = 8, *Fstl1*^−/−^, *n* = 3; E12.5: *Fstl1*^*+/+*^, *n* = 13, *Fstl1*^−/−^, *n* = 9; E13.5: *Fstl1*^*+/+*^, *n* = 21, *Fstl1*^−/−^, *n* = 6; E15.5: *Fstl1*^*+/+*^, *n* = 11, *Fstl1*^−/−^, *n* = 5; E18.5: *Fstl1*^*+/+*^, *n* = 5, *Fstl1*^−/−^, *n* = 3. ∗, *p* < 0.05; ∗∗∗∗, *p* < 0.0001*.* (F) Quantification of tracheal tube length after whole-mount staining. E11.5: *Fstl1*^*+/+*^, *n* = 6, *Fstl1*^−/−^, *n* = 4; E12.5: *Fstl1*^*+/+*^, *n* = 11, *Fstl1*^−/−^, *n* = 7; E13.5: *Fstl1*^*+/+*^, *n* = 16, *Fstl1*^−/−^, *n* = 5; E15.5: *Fstl1*^*+/+*^, *n* = 7, *Fstl1*^−/−^, *n* = 4; E18.5: *Fstl1*^*+/+*^, *n* = 4, *Fstl1*^−/−^, *n* = 3. Data are expressed as the mean ± SEM. Differences between the groups were assessed using Student’s *t* tests.

We have previously shown that *Fstl1*^−/−^ neonates have unusually enlarged tracheal tubes with irregular inner margins.^11^ To examine the defects of luminal epithelial morphogenesis in *Fstl1*^−/−^ tracheas in detail, we collected embryos from the elongation/expansion stage at indicated developmental days and performed histological analysis on the transverse sections from the middle level of tracheas. We found that the enlarged lumen size in *Fstl1*^−/−^ tracheas was evident as early as E11.5 and that this enlargement continued to increase significantly along with the tracheal development, until postnatal day 0 (P0) (Figures 1A and S1A). Quantitative analysis confirmed the larger lumen size in *Fstl1*^−/−^ tracheas than that of age-matched wild type (WT, *Fstl1*^*+/+*^) littermates at all indicated developmental days, as determined by tracheal lumen circumference and area (Figures 1B and 1C; Figures S1B and S1C). We also isolated embryonic tracheas with lung tissues (E11.5–E18.5) and performed whole-mount immunofluorescence staining for the epithelial marker E-cadherin. This staining highlighted epithelial cells lining the tracheal lumina, providing a clear visualization of the lumens (Figure 1D). Consistent with the above histological findings, *Fstl1*^−/−^ tracheas displayed enlargement of the lumens compared with those of the age-matched *Fstl1*^*+/+*^ littermates, as determined by tracheal lumen diameters (Figures 1D and 1E). We also measured their length and found that it was comparable between *Fstl1*^*+/+*^ and *Fstl1*^−/−^ tracheas at all stages examined (Figure 1F).

Of note, both *Fstl1*^*+/+*^ and *Fstl1*^−/−^ tracheas (E11.5–E13.5) exhibited a round tubular shape lined by a smooth epithelial lumen. However, starting from E15.5, we observed that the tracheal tubes of *Fstl1*^−/−^ embryos were deformed. At E18.5 and P0, the tracheal tubes were severely deformed, and their luminal epithelial layers were irregular with folds and protuberances (Figures 1A and S1A), as previously reported.^11^ These findings indicate an atelectatic phenotype of *Fstl1*^−/−^ tracheas at the late stage of development due to the insufficient support from truncated cartilage rings and impaired SM.^12^ This atelectatic phenotype became more severe at birth (P0), as indicated by the increased lumen circumference but comparable lumen area in *Fstl1*^−/−^ tracheas (Figures S1A–S1C).

We also examined the lumen size of the main bronchi. Histological analysis was conducted on transverse sections of the trachea, bifurcation sites, and main bronchi from E12.5 embryos (Figures S1D and S1E). Consistently, the lumens of the *Fstl1*^−/−^ trachea, bifurcation, and both left and right bronchi were significantly enlarged. This lumen enlargement was further validated through quantitative measurements of the lumen circumference and area (Figures S1F and S1G).

### Increased cell proliferation in *Fstl1*^−/−^ tracheal epithelium

During early development of the trachea, the luminal epithelial morphogenesis mainly relies on cell proliferation.^3^ To examine whether the enlarged lumen in *Fstl1*^−/−^ tracheas is associated with the negative regulation of Fstl1 on epithelial proliferation,^11^ we conducted bromodeoxyuridine (BrdU) incorporation assay. As shown in Figures S2A and S2B, cell proliferation increased along with the tube elongation/expansion, reaching the peak at E13.5 and decreasing at E15.5. This is in agreement with the recent report that tracheal epithelial proliferation decreases after E14.5.^3^ As expected, *Fstl1* deletion increased cell proliferation during all developmental stages in *Fstl1*^−/−^ tracheal epithelium, as quantitated by the percentages of BrdU^+^ cells at indicated developmental days (Figures S2A and S2B).

After E14.5, when cell proliferation decreased, the luminal size increased by cell rearrangement, expansion of the apical surface, and modest cell proliferation.^3^^,^^4^ To examine whether changed cell rearrangement also contributed to the lumen enlargement of *Fstl1*^−/−^ tracheas after E14.5, we measured the apical surface of luminal epithelial cells (E18.5). Integration of quantitative values confirmed the modest increases in cell numbers and significant increases in apical surface in *Fstl1*^−/−^ tracheal epithelium (Figures S2C–S2E).

### Transverse orientation in dividing tracheal epithelial cells

**Figure 2:**
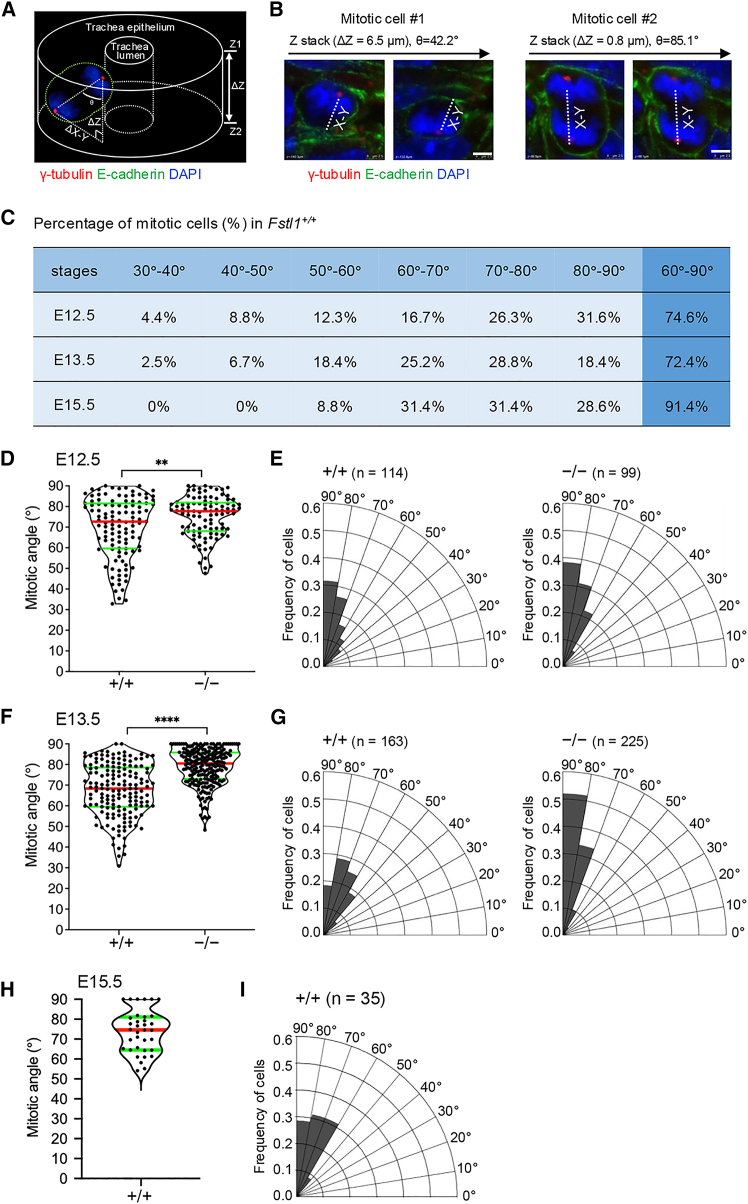
Increased mitotic spindle angles in tracheal epithelial cells of the *Fstl1*-deficient embryos (A) Schematic graph of the mitotic spindle angles in the tracheal epithelial cells. (B) Representative cells with different mitotic angles. Immunostaining for centrosomes (γ-tubulin), epithelial cells (E-cadherin), and nuclei (DAPI) was performed to determine the mitotic spindle angles of the tracheal epithelial cells. Scale bars: 2.5 μm. (C) Data chart of the percentage change of mitotic spindle angles at E12.5, E13.5, and E15.5 in *Fstl1*^*+/+*^ tracheal epithelial cells. (D and E) Violin plots (D) and Rose plots (E) of mitotic spindle angles of tracheal epithelial cells at E12.5 in *Fstl1*^*+/+*^ and *Fstl1*^−/−^ mouse embryos. *Fstl1*^*+/+*^, *n* = 114; *Fstl1*^−/−^, *n* = 99. ∗∗, *p* < 0.01*.* (F and G) Violin plots (F) and Rose plots (G) of mitotic spindle angles of tracheal epithelial cells at E13.5 in *Fstl1*^*+/+*^ and *Fstl1*^−/−^ mouse embryos. *Fstl1*^*+/+*^, *n* = 163; *Fstl1*^−/−^, *n* = 225. ∗∗∗∗, *p* < 0.0001. (H and I) Violin plots (H) and Rose plots (I) of mitotic spindle angles of tracheal epithelial cells at E15.5 in *Fstl1*^*+/+*^ mouse embryos. *Fstl1*^*+/+*^, *n* = 35. Data are expressed as the mean ± SEM. Differences between the groups were assessed using Student’s *t* tests.

The morphogenesis and maintenance of tubular organs rely on the proper orientation of the mitotic spindle during cell division.^13^ To identify oriented cell division of the tracheal epithelial cells and to address whether changes in their mitotic spindle angles also account for the enlarged lumen in *Fstl1*^−/−^ trachea, we adapted a geometric model based on a previously reported approach^9^ and measured the spindle angles in individual mitotic cells. In this model, the mitotic angles of tracheal epithelial cells were measured by the distances between the mitotic spindle poles both perpendicular and parallel to the tracheal longitudinal axis. Epithelial cells were identified via E-cadherin staining, and mitotic spindle poles were marked using γ-tubulin. z stacks of spindle poles in dividing cells at anaphase and telophase were acquired using Leica TCS SP5 and Zeiss LSM710 confocal microscopes. The distance parallel to the tracheal longitudinal axis (Δ*z*) was calculated from the two z stacks, and the confocal images from these z stacks were merged to calculate the distance perpendicular to the longitudinal axis (Δ*x* − *y*), using ImageJ software. The mitotic angle (θ) was then computed using the formula: θ = ATAN[(Δ*x* − *y*)/Δ*z*] × 180/π (Figure 2A). Smaller mitotic angles (θ) were associated with biased contribution to tracheal length, while larger mitotic angles contributed to tracheal circumference (Figure 2B).

We first measured the mitotic spindle orientation of dividing epithelial cells in *Fstl1*^*+/+*^ tracheas at indicated developmental days. Unlike the longitudinal orientation in lung epithelium,^9^ a large proportion of mitotic cells (E12.5, 74.6%; E13.5, 72.4%; E15.5, 91.4% of mitotic cells) in tracheal epithelium had their spindles tending to be vertical to the tracheal longitudinal axis (θ = 60°–90°) (Figures 2C–2I). These data indicated that the transverse orientation determines the wide lumen morphogenesis of tracheal tube while elongating along the AP axis.

### Abnormal spindle angle distribution in proliferating cells of *Fstl1*^−/−^ tracheal epithelium

To address whether changes of mitotic spindle angle in the epithelial cells account for the enlarged lumen in *Fstl1*^−/−^ trachea, we next measured spindle angles in *Fstl1*^−/−^ tracheas. Because of the deformed tubular shapes at late stages (after E15.5), E12.5 and E13.5 *Fstl1*^−/−^ tracheas with round tube shape were used to study changes in the oriented cell division. In E12.5 *Fstl1*^−/−^ tracheal epithelium, more mitotic cells had their spindles vertical to the tracheal longitudinal axis than those in *Fstl1*^*+/+*^ (θ peaks at 70°–90°; *Fstl1*^*+/+*^, 57.9%; *Fstl1*^−/−^, 69.7%) (Figures 2D and 2E). This bias was more pronounced in E13.5 *Fstl1*^−/−^ tracheal epithelium, with a higher proportion of mitotic cells and more vertical spindle angles (*Fstl1*^*+/+*^, θ peaks at 60°–80°, 54.3%; *Fstl1*^−/−^, θ peaks at 70°–90°, 85.8%) (Figures 2F and 2G). These data highlighted a clear shift in spindle orientation distribution associated with *Fstl1* deficiency.

### Comparable ERK1/2 signaling in *Fstl1*^−/−^ tracheal epithelial cells

RAS-regulated ERK1/2 signaling has been reported to control the mitotic spindle angles of airway epithelial cells in the lungs.^9^ To investigate the molecular mechanisms whereby the deficiency of *Fstl1* results in lumen enlargement of mouse trachea described above, we first examined the ERK1/2 signaling activity. Immunostaining for *p*-ERK1/2 on the transverse sections of E12.5 and E13.5 embryos showed comparable ERK1/2 activity in *Fstl1*^*+/+*^ and *Fstl1*^−/−^ tracheal epithelial cells (Figure S3A). In addition, *in vivo* inhibition of the ERK1/2 pathway using the specific inhibitor PD0325901 failed to rescue the enlarged tracheal lumen in E12.5 and E13.5 *Fstl1*^−/−^ embryos (Figures S3B and S3C).

### Ectopic activation of BMP4 signaling in *Fstl1*^−/−^ tracheal epithelium

**Figure 3:**
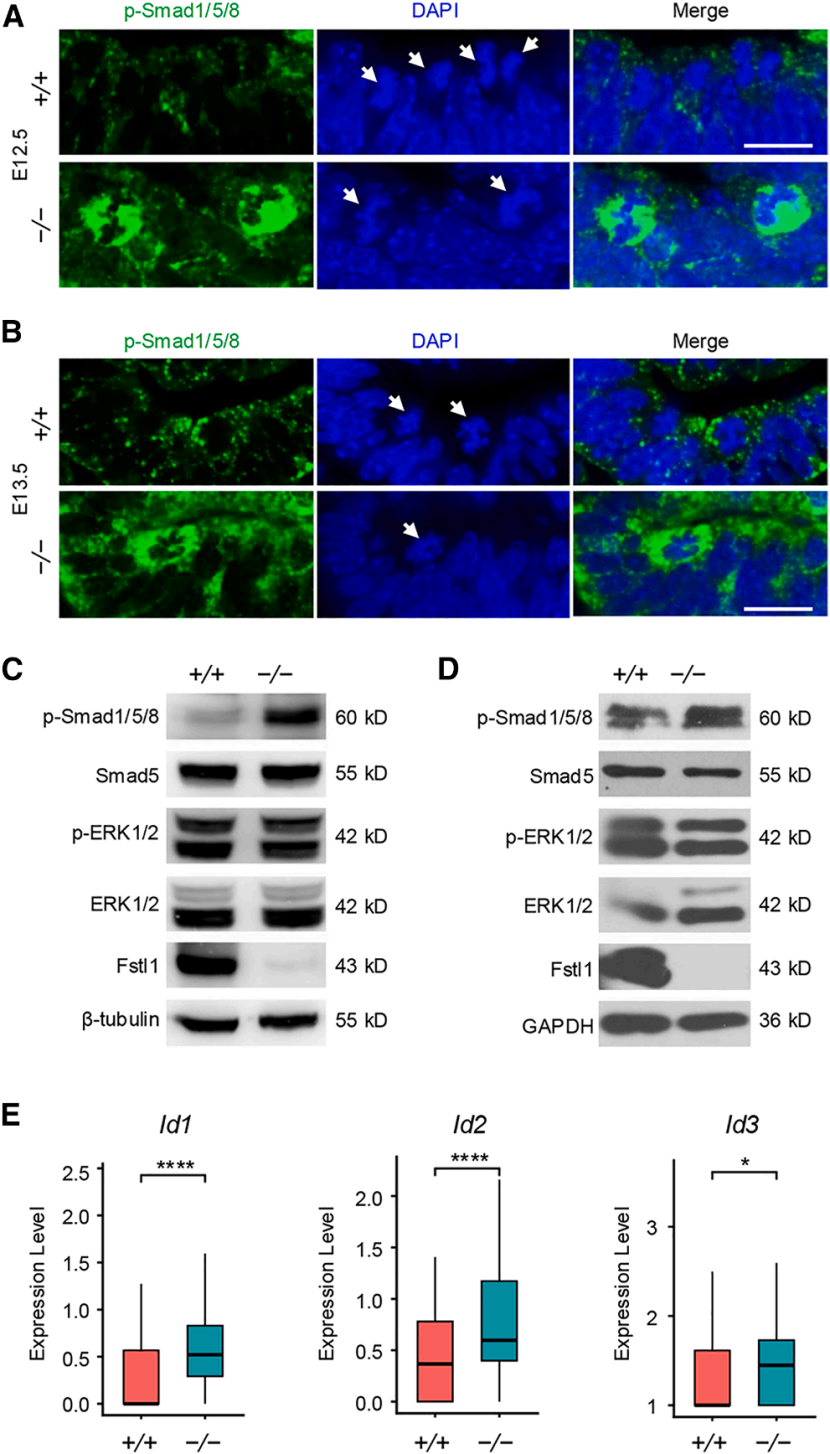
Ectopic activation of BMP4 signaling in tracheal epithelial cells of the *Fstl1*-deficient embryos (A and B) Immunostaining for *p*-Smad1/5/8 was performed on E12.5 (A) and E13.5 (B) in *Fstl1*^*+/+*^ and *Fstl1*^−/−^ mouse tracheal sections. *n* = 3. Arrows indicate the proliferating epithelial cells. Scale bars: 10 μm. (C and D) Western blot for *p*-Smad1/5/8 and *p*-ERK1/2 in E16.5 (C) and E18.5 (D) *Fstl1*^*+/+*^ and *Fstl1*^−/−^ mouse tracheal tissues. Data are representative of two independent experiments. (E) Bar plots showing the expression of canonical BMP4 target genes *Id1*, *Id2*, and *Id3* in Epcam^+^Sox2^+^ proximal airway epithelial cells from scRNA-seq in E18.5 *Fstl1*^*+/+*^ and *Fstl1*^−/−^ lungs (GSE225463). Data are expressed as the mean ± SEM. Differences between the groups were assessed using Student’s *t* tests.

Because Fstl1 acts as a BMP4 antagonist in lung and ureter development,^11^^,^^14^ we next examined BMP4 signaling activity. To our surprise, *Fstl1*^−/−^ tracheal epithelial cells, particularly the proliferating cells, exhibited significantly elevated immunostaining signals of *p*-Smad1/5/8, a reliable marker of BMP4 canonical signaling activity, in both E12.5 (Figure 3A) and E13.5 (Figure 3B) embryos. Western blot analysis using total protein extracts from E16.5 (Figure 3C) and E18.5 (Figure 3D) tracheal tissues further confirmed higher levels of *p*-Smad1/5/8, whereas *p*-ERK1/2 levels remained unchanged (Figures 3C and 3D) in *Fstl1*^−/−^ tracheas. We also identified proximal airway epithelial cells (Epcam^+^Sox2^+^) from our previously published single-cell RNA sequencing (scRNA-seq) dataset on E18.5 mouse lungs.^15^ Bar plots further confirmed that the expression of BMP4 signaling downstream targets (*Id1*, *Id2*, and *Id3*) were markedly upregulated in the proximal airway epithelial cells from *Fstl1*^−/−^ lungs compared with those from *Fstl1*^*+/+*^ lungs (Figure 3E). These findings indicated that BMP4 signaling, rather than ERK1/2 signaling, is aberrantly activated in *Fstl1*^−/−^ tracheal epithelial cells, potentially contributing to abnormal orientated cell division and the enlarged tracheal lumen phenotype.

### Comparable Wnt5a-Ror2 signaling pathway in *Fstl1*^−/−^ tracheas

Multiple signaling pathways, including the mesenchymal Wnt5a-Ror2 axis,^4^ have been implicated in tracheal epithelial polarization and tube morphogenesis. To determine whether this pathway contributes to the defects observed in *Fstl1*^−/−^ tracheas, we examined its activity at both protein and transcript levels. Western blot analysis of E16.5 and E18.5 tracheal tissues (Figures S4A and S4B) showed no significant changes in Wnt5a or Ror2 expression in *Fstl1*^−/−^ samples. Consistently, expression analysis of E18.5 tracheas (Figure S4C) revealed no significant differences in *Wnt5a*, *Ror2*, or the downstream target *Axin2* between *Fstl1*^*+/+*^ and *Fstl1*^−/−^ tracheas. These results suggest that the Wnt5a-Ror2 signaling pathway is not significantly altered in the absence of Fstl1 and is unlikely to account for the observed phenotypes.

### Blocking BMP4 signaling activity rescues lumen enlargement in *Fstl1*^−/−^ tracheas

**Figure 4:**
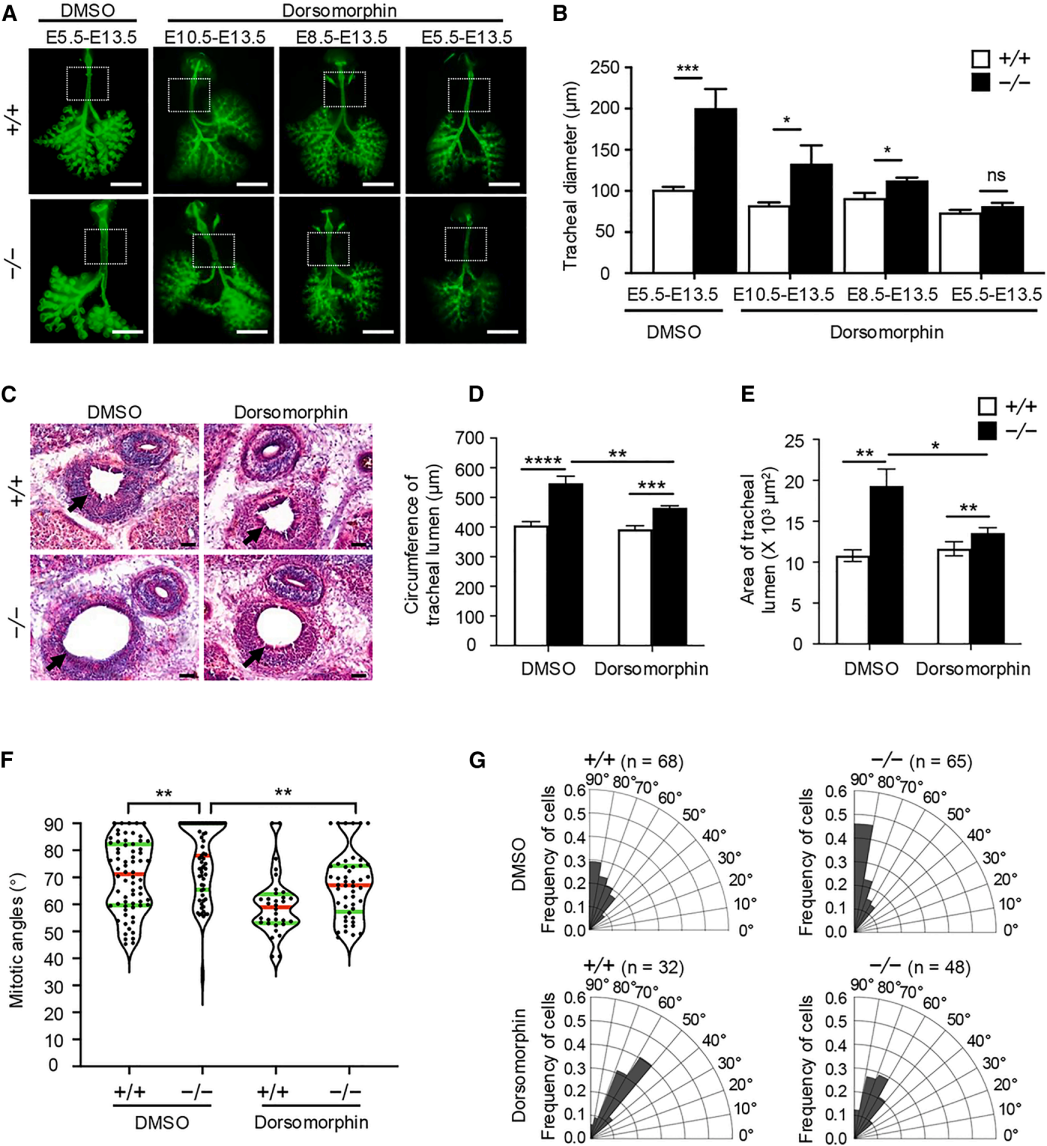
Blocking BMP4 signaling rescued enlarged tracheal lumen in *Fstl1*-deficient embryos (A and B) Whole-mount staining of E-cadherin (A) and quantification of the tracheal lumen diameters (B) in *Fstl1*^*+/+*^ and *Fstl1*^−/−^ mouse tracheas after different doses of DMSO or dorsomorphin treatment *in vivo*. DMSO: *Fstl1*^*+/+*^, *n* = 7, *Fstl1*^−/−^, *n* = 4; dorsomorphin: E10.5–E13.5, *Fstl1*^*+/+*^, *n* = 5, *Fstl1*^−/−^, *n* = 4; E8.5–E13.5, *Fstl1*^*+/+*^, *n* = 4, *Fstl1*^−/−^, *n* = 4; E5.5–E13.5, *Fstl1*^*+/+*^, *n* = 4, *Fstl1*^−/−^, *n* = 3. Scale bars: 500 μm ∗, *p* < 0.05; ∗∗∗, *p* < 0.001. Differences between the groups were assessed using Student’s *t* tests. (C–E) Representative images of H&E staining (C), and the quantification of tracheal lumen circumference (D) and area (E) at E13.5 in *Fstl1*^*+/+*^ and *Fstl1*^−/−^ mouse embryos with daily DMSO or dorsomorphin treatment *in vivo* from E5.5 to E13.5. *n* = 6. Scale bars, 100 μm ∗, *p* < 0.05; ∗∗, *p* < 0.01; ∗∗∗, *p* < 0.001; ∗∗∗∗, *p* < 0.0001. Differences between the groups were assessed using one-way ANOVA. (F and G) Violin plots (F) and Rose plots (G) of mitotic spindle angles of tracheal epithelial cells at E13.5 in *Fstl1*^*+/+*^ and *Fstl1*^−/−^ mouse embryos with daily DMSO or dorsomorphin treatment *in vivo* from E5.5 to E13.5. ∗∗, *p* < 0.01. Differences between the groups were assessed using one-way ANOVA. Data are expressed as the mean ± SEM.

To test if the activated BMP4/Smad signaling plays a causative role in the development of lumen enlargement in *Fstl1*^−/−^ tracheas, we injected pregnant mice with different doses of dorsomorphin, a selective BMP4 signaling inhibitor, at the indicated time points, and isolated E13.5 embryonic tracheas for whole-mount E-cadherin immunostaining. As shown in Figures 4A and 4B, *Fstl1*^−/−^ tracheas displayed the phenotype of lumen enlargement with the tube diameter 97.5% wider than that of *Fstl1*^*+/+*^ controls. By contrast, dorsomorphin injections decreased the tracheal lumen size of *Fstl1*^−/−^ embryos, with the tracheal lumen being 61.4% wider than those of *Fstl1*^*+/+*^ controls at 3 doses from E10.5 to E13.5 and 23.4% wider at 5 doses from E8.5 to E13.5, while 8 doses from E5.5 to E13.5 almost restored the tracheal lumen to the normal size. These findings indicated that the abnormal lumen enlargement seen in *Fstl1*^−/−^ embryos was partially rescued by BMP4/Smad signaling inhibition. Histological examination of E13.5 embryos treated with 8 doses of dorsomorphin confirmed the partially rescued lumen enlargement in *Fstl1*^−/−^ embryos, as determined by the decreased tracheal lumen circumference and area (Figures 4C–4E). Violin plots and Rose plots further confirmed a smaller proportion of mitotic cells with smaller vertical spindle angles (*Fstl1*^−/−^, 69.3% cells with θ peaking at 70°–90°; *Fstl1*^−/−^ + dorsomorphin, 56.3% cells with θ peaking at 60°–80°) in tracheal epithelial cells of E13.5 *Fstl1*^−/−^ embryos administered with 8 doses of dorsomorphin compared with *Fstl1*^*+/+*^ controls (44.1% cells with θ peaking at 60°–80°) (Figures 4F and 4G; Figure S5A).

In addition, we employed an explant culture model with E12.5 lung,^16^ and treated the cultures with dorsomorphin to block BMP4 signaling activity. After *ex vivo* culture for 48 h, dorsomorphin treatment decreased the diameter of *Fstl1*^−/−^ tracheal tubes, resulting in comparable tracheal tube size between *Fstl1*^−/−^ and *Fstl1*^*+/+*^ controls (Figures S5B and S5C). Collectively, Fstl1 negatively regulates BMP4/Smad signaling, which controls oriented cell division and consequently determines the lumen size of the tracheal tube.

### BMP4/Smad signaling influences spindle orientation in BEAS-2B cells

To further examine the role of BMP4 signaling in regulating spindle orientation in tracheal epithelial cells, we treated BEAS-2B cells, a human proximal bronchial epithelial cell line, with a recombinant human BMP4 protein (20 ng/mL). BMP4 stimulation significantly activated BMP4 signaling but not ERK1/2 signaling, as determined by the increased levels of *p*-Smad1/5/8 but comparable *p*-ERK1/2 (Figure S6A), as well as elevation of BMP4 downstream genes (Figure S6B). We calculated the mitotic angle (α) of epithelial cells based on the vertical distance (Δ*z*) and horizontal distance (10) between the mitotic spindle poles labeled with γ-tubulin, using the formula α = ATAN(Δ*z*/*x*) × 180/π (Figure S6C). As shown in Figures S6D and S6E, compared with a large proportion of BEAS-2B cells (92%) dividing parallel to the horizontal axis (α peaks at 0°–10°), BMP4 treatment significantly disrupted the bias, and spindle orientation distribution shifted to a higher angular range (α peaks at 0°–50°).

To determine whether the role of BMP4 signaling-regulated spindle orientation is specific to the tracheal epithelial cells, we extended our analysis to MLE12 cells, a mouse alveolar epithelial cell line, following BMP4 treatment. As shown in Figures S6F and S6G, BMP4 did not induce any changes in the mitotic spindle orientation in MLE12 cells. These results suggest that the above mechanism is specific to the tracheal epithelium and does not extend to the lung alveolar epithelial cells.

### Fstl1-downregulated BMP4/Smad signaling influences spindle orientation in BEAS-2B cells

Fstl1 is a mesenchymal-derived matricellular protein. Using a Fstl1 expression reporter mouse line (*Fstl1*-LacZ), we have previously shown a clear Fstl1 expression in subepithelial mesenchymal cells surrounding the tracheal epithelium (E18.5).^12^ To further characterize the expression pattern of Fstl1 during early lung development, we performed an integrative analysis of published scRNA-seq datasets from E8.0–E9.5 mouse foregut^17^ and E9.5–E11.5^18^ and E12.5^19^ mouse embryonic lungs. Comparative analysis revealed that *Fstl1* exhibited a high but dynamic spatial-temporal expression pattern in the mesoderm/mesenchymal compartment. In contrast, several other BMP4 antagonists, including Nog, Fst, Chrd, Grem1, and Sostdc1, showed comparatively lower expression levels during these stages (Figure S7A), suggesting a predominant role for Fstl1 in modulating BMP4 signaling in lung mesenchyme during early development.

**Figure 5:**
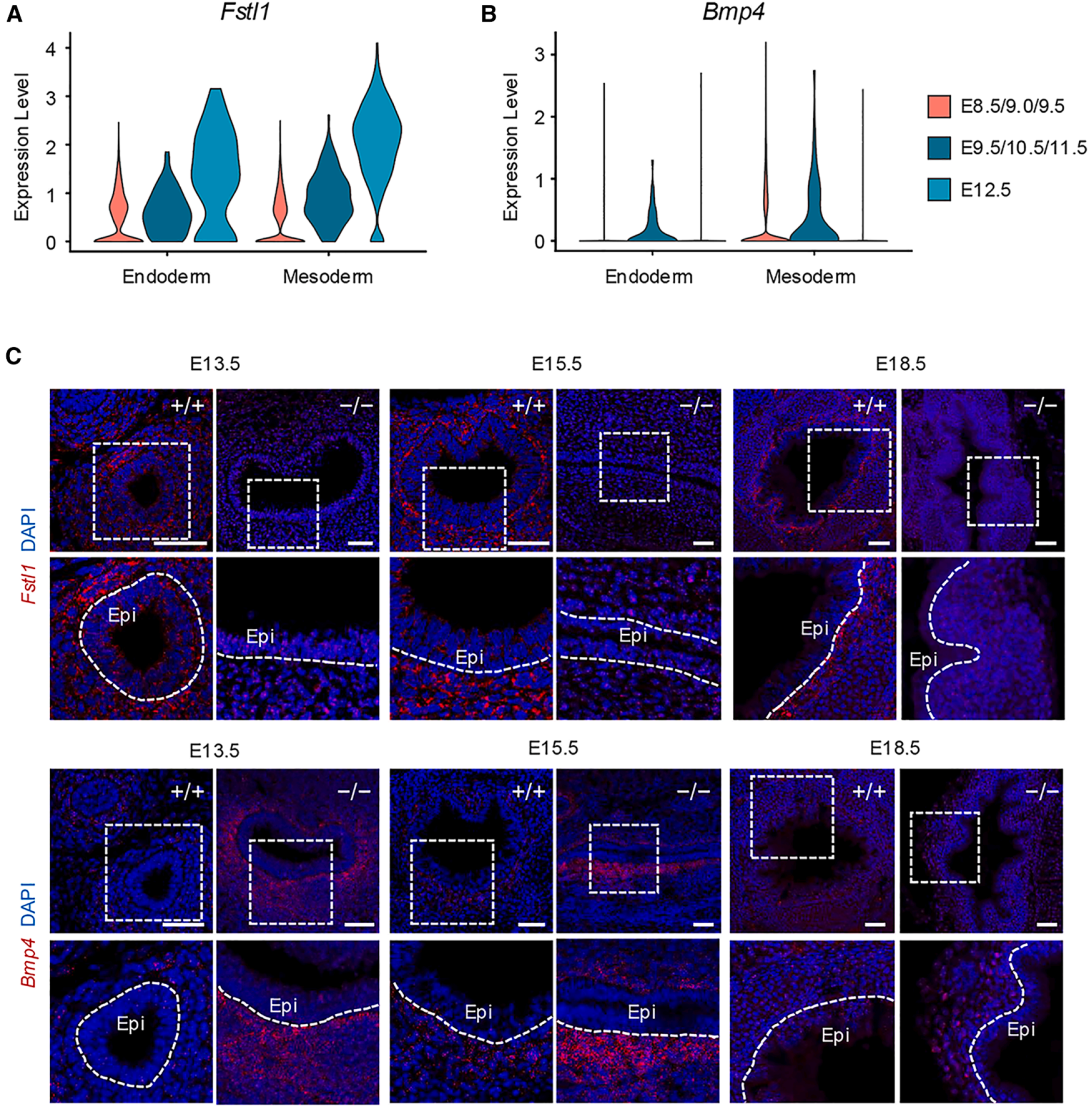
BMP4 signaling is downregulated because of the expression of Fstl1 in the tracheal mesenchyme (A and B) Violin plots showing the expression of *Fstl1* (A) and *Bmp4* (B) in published scRNA-seq data on mouse E8.5/9.0/9.5 foregut (GSE136689), E9.5/10.5/11.5 (GSE87038), and E12.5 (GSE119228) lungs. (C) RNAscope *in situ* hybridization of *Fstl1* and *Bmp4* on tracheal transverse sections in *Fstl1*^*+/+*^ and *Fstl1*^−/−^ mouse embryos at E13.5, E15.5, and E18.5. Dashed boxes mark regions displayed at a higher magnification, and dashed lines delineate the boundary between the epithelium and mesenchyme. Scale bars: 50 μm.

We next compared the spatial and temporal expression patterns of *Fstl1* and *Bmp4*. As shown in Figures 5A and 5B, *Fstl1* expression is generalized in both mesoderm and endoderm of the foregut but becomes highly enriched in the mesoderm in later tracheal formation stages, whereas *Bmp4* transcripts are localized mainly in the mesoderm, with little expression detected in the endoderm. Of note, after E11.5, we observed a significant increase in *Fstl1* expression but a remarkable decrease in *Bmp4* expression (Figures 5A and 5B). To further validate these observations in the trachea, RNAscope analysis of E13.5, E15.5, and E18.5 sections confirmed that *Fstl1* is primarily localized to the mesenchymal cells surrounding the airway epithelium. Importantly, loss of *Fstl1* led to a marked increase in *Bmp4* transcript levels within the tracheal mesenchyme of *Fstl1*^−/−^ embryos (Figure 5C), supporting a regulatory relationship between Fstl1 and Bmp4 signaling *in vivo*.

**Figure 6:**
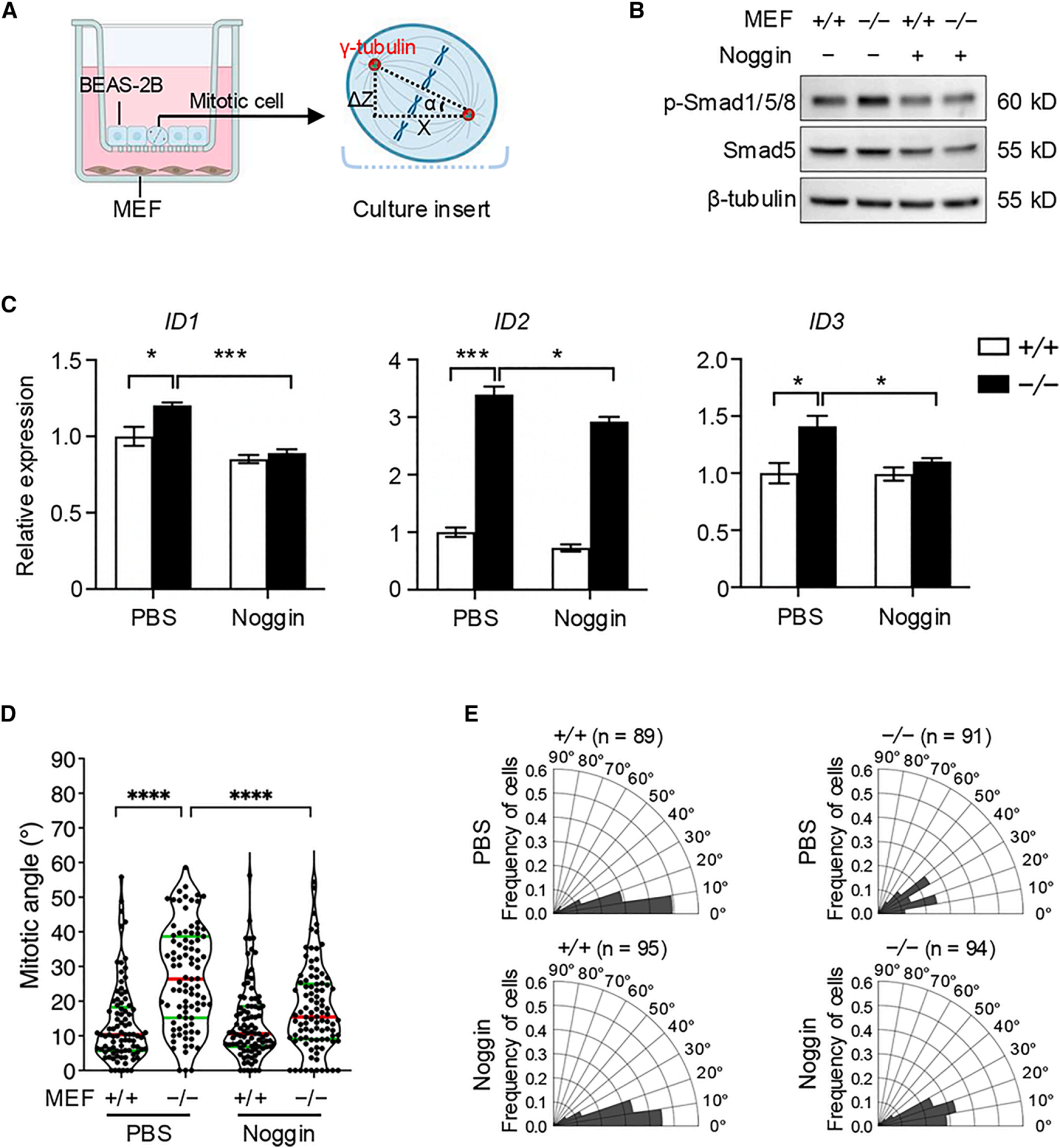
Fstl1-downregulated BMP4/Smad signaling influences spindle orientation in BEAS-2B cells (A) Schematic graph of the BEAS-2B cells and MEF co-culture system and the mitotic spindle angles of BEAS-2B cells. (B) Western blot analysis of *p*-Smad1/5/8 and Smad5 in BEAS-2B cells co-cultured with *Fstl1*^*+/+*^ and *Fstl1*^−/−^ MEFs with PBS or Noggin (25 ng/mL). Data are representative of three independent experiments. (C) BEAS-2B cells were co-cultured with *Fstl1*^*+/+*^ and *Fstl1*^−/−^ MEFs and treated with PBS or Noggin (25 ng/mL), and relative expression of *ID1*, *ID2*, and *ID3* was determined by RT-qPCR. *n* = 3. ∗, *p* < 0.05; ∗∗∗, *p* < 0.001. (D and E) Violin plots (D) and Rose plots (E) showing the mitotic spindle angles of BEAS-2B cells co-cultured with *Fstl1*^*+/+*^ and *Fstl1*^−/−^ MEFs and treated with PBS or Noggin (25 ng/mL). PBS: *Fstl1*^*+/+*^, *n* = 89, *Fstl1*^−/−^, *n* = 91; Noggin: *Fstl1*^*+/+*^, *n* = 95, *Fstl1*^−/−^, *n* = 94. ∗∗∗∗, *p* < *0.0001*. Data are expressed as the mean ± SEM. Differences between the groups were assessed using one-way ANOVA.

To further validate the role of Fstl1 in regulating epithelial mitotic spindle orientation through the BMP4/SMAD signaling pathway, we isolated mouse embryonic fibroblasts (MEFs) from E13.5 *Fstl1*^−/−^ embryos. These cells showed a marked reduction in Fstl1 expression and secretion (Figures S7B and S7C), accompanied by an increase in Bmp4 expression and secretion (Figures S7D–S7F). We then employed a transwell co-culture system with BEAS-2B epithelial cells in the upper chamber and MEFs in the lower plate (Figure 6A). To assess the specificity of BMP4 signaling, we also included Noggin, a well-characterized BMP4 antagonist, as an additional control. As expected, we found that co-culture with *Fstl1*^−/−^ MEFs increased the level of *p*-Smad1/5/8 and expression of BMP4 target genes (*ID1*, *ID2*, and *ID3*) in BEAS-2B cells (Figures 6B and 6C), and subsequently increased the proportion of cells with mitotic angle distributing to the angular range (α peaks at 0°–60°). Treatment with Noggin decreased the levels of *p*-Smad1/5/8 and the proportion of cells skewed toward low angles (Figures 6D and 6E). These findings establish a functional role for Fstl1 in regulating epithelial BMP4/Smad1/5/8 signaling and mitotic angles in epithelial cells, thereby influencing tracheal shape formation in mice.

## Discussion

Understanding the mechanisms underlying tube formation is a fundamental goal in developmental biology. The reported roles of epithelial cells in the development of the wide lumen of tracheal tubes correlate with their proliferation, shape change, and arrangement.^3^^,^^4^ The findings reported here add new insights into our understanding of tracheal tubulogenesis and demonstrate the oriented epithelial division may influence the size of the tracheal lumen. We provide data from knockout mouse models, *in vivo* pharmacological intervention, *in vitro* co-culture system, as well as single-cell transcriptome analysis to support the role of Fstl1-regulated Bmp4/Smad signaling axis in the determination of the oriented epithelial division and subsequent tracheal luminal growth.

Although the luminal epithelial morphogenesis of the trachea and lungs mainly relies on cell proliferation, the regulatory mechanisms are not the same. In the lungs, the dividing cells are oriented longitudinally by default, with a large proportion of epithelial cells dividing parallel to the airway longitudinal axis, allowing the tube length to increase more than circumference as it grows along the AP axis at the early stage.^9^ In contrast, Kishimoto et al. observed that the epithelial cells of the trachea exhibit random orientations.^3^^,^^4^ Here, we adopted the geometric model as reported by Tang et al.^9^ and marked the mitotic spindle poles by using γ-tubulin, instead of using nuclei. We measured the mitotic spindle angle of the dividing tracheal epithelial cells at different developmental days (E12.5, E13.5, and E15.5) and showed that tracheal epithelial cells are oriented and that most mitotic cells divide with their spindles nearly vertical to the tracheal longitudinal axis. Their transverse orientation shapes the development of the wide lumen of the tracheal tube as it extends along the AP axis. We further showed that this process involves canonical BMP4/Smad1/5/8 signaling, distinguishing it from lung airway tube morphogenesis, in which RAS/ERK1/2 signaling determines longitudinal orientation.^9^ Altogether, our data provide evidence to support that lumen epithelial morphogenesis of the trachea and lungs is quite different.

Fstl1, an antagonist for BMP4 signaling, has been reported in alveologenesis and regeneration of alveolar epithelium from alveolar type 2 (AT2) stem cells.^11^^,^^20^ Here, we offer the following lines of evidence to demonstrate the critical role of the BMP4 antagonist Fstl1 in determining luminal epithelial morphogenesis of trachea: targeting deletion of *Fstl1* in mice leads to tracheal lumen size to grow wider; Fstl1 exerts its effect on luminal size through the regulation of oriented cell division, together with cell proliferation and cell shape. Fstl1-reduced BMP4/Smad signaling primes or allows tracheal epithelial cells to divide with transverse orientation, determining the development of the wide lumen of the tracheal tube. Pharmacological intervention on BMP4 signaling with dorsomorphin partially recovers the tracheal lumen size of *Fstl1*^−/−^ embryos and mitotic spindle angle *in vivo* and *ex vivo*.

BMP4 signaling has been implicated in the tubulogenesis of trachea, especially dorsal-ventral (DV) pattern establishment and tracheal lineage specification.^3^^,^^7^ In the current mouse model, we observed the upregulation of phospho-Smad1/5/8, together with the increased expressions of *Id1*, *Id2* and *Id3—*established BMP/SMAD downstream targets*—*in the wider *Fstl1*^−/−^ luminal epithelium. Reducing BMP4 signaling activity by Noggin partially rescued the mitotic spindle angle and reversed the increased *ID1*, *ID2*, and *ID3* expressions in transwell-cultured BEAS-2B cells *in vitro*. Together with the *in vivo* pharmacological intervention data, the findings support our hypothesis that the BMP4 signaling axis is crucial for luminal epithelial morphogenesis and that Fstl1 affects oriented cell division and subsequent lumen growth in the developing trachea partially through modulating the BMP4 signaling axis. Although multiple signaling pathways have been implicated in the development of trachea, no evidence of the interaction between Fstl1 and ERK1/2 signaling pathway or Fstl1 and Wnt5a-Ror2 signaling pathway was found in our *Fstl1*^−/−^ embryos.

Fstl1 may orchestrate with other BMP antagonists via their individual spatiotemporal expression patterns and control BMP4 signaling in the developing trachea. BMP4 signaling is downregulated in the dorsal foregut because of the expression of Noggin in the dorsal endoderm foregut in early embryo.^3^ Fstl1 starts to be expressed at E7.^21^ Its expression level increases during the development of trachea, being ubiquitous until E14.5 and restricted to the mesenchyme surrounding the tracheal tube at later stages.^3^ Single-cell transcriptome analyses indicate a mutually exclusive spatiotemporal expression pattern between Noggin and Fstl1, indicating the balance of BMP4 signaling by different antagonists temporally, with Noggin being predominant in stage of DV patterning (E8.5–E10.5), whereas Fstl1 being pronounced in the tube elongation/expansion stage (E10.5–18.5). Almost no expression of other BMP4 antagonists, including Fst, Grem1, and Chrd, in trachea further suggests the importance of Fstl1 on BMP4 signaling regulation in the developing trachea.

In summary, our findings reveal a previously unrecognized mechanism underlying tracheal tubulogenesis, demonstrating that oriented epithelial cell division plays a critical role in determining the luminal size. We show that, unlike in the lung, tracheal epithelial cells predominantly divide with a transverse orientation, contributing to lumen widening along the AP axis. Importantly, we identify the Fstl1-BMP4/Smad signaling axis as a key regulator of this process, integrating control of mitotic orientation with epithelial proliferation and cell shape. Through complementary *in vivo* and *in vitro* approaches, we establish that Fstl1 modulates BMP4 signaling to ensure proper tracheal epithelial morphogenesis and that the disruption of this balance leads to aberrant lumen expansion. Our work not only distinguishes the mechanisms governing tracheal versus lung development but also highlights a novel functional role for BMP4 antagonism in directing oriented cell division. These insights advance our understanding of early tracheal development and provide a conceptual framework for how signaling pathways coordinate epithelial architecture during organogenesis.

### Limitations of the study

In this study, we report that Fstl1 negatively regulates BMP4/Smad1/5/8 signaling and that the deficiency of *Fstl1* leads to abnormal lumen enlargement and disrupted tube morphology. Fstl1 is a matricellular protein produced mainly by cells of mesenchymal origin.^12^^,^^21^^,^^22^^,^^23^ We have previously shown the heterogeneity of Fstl1 expression in multiple mesenchymal subpopulations in E18.5 lungs by scRNA-seq analysis,^15^ but it remains to be clarified which mesenchymal subpopulations regulate the morphogenesis of trachea. We will use genetic lineage tracing, RNA-scope and scRNA-seq approaches to explore this issue.

## Resource availability

### Lead contact

Requests for further information and resources should be directed to and will be fulfilled by the lead contact, Wen Ning (ningwen108@nankai.edu.cn).

### Materials availability

This study did not generate new unique reagents.

### Data and code availability

• This paper analyzes existing, publicly available data, accessible at GEO: GSE136689, GSE87038, GSE119228, and GSE225463.
• This paper does not report original code.
• Original western blotting images have been deposited at supplemental PDF and are publicly available as of the date of publication. Microscopy data reported in this paper will be shared by the lead contact upon request.
• Any additional information required to reanalyze the data reported in this paper is available from the lead contact upon request.

## Acknowledgments

We thank Nan Tang and Jun Zhou for techniques assistance in the determination of mitotic spindle angles. This work was supported by 10.13039/501100012166National Key Research and Development Program of China grant 2024YFA1108500 and 10.13039/100014718National Natural Science Foundation of China (NSFC) grants 82030001 and 31271559.

## Author contributions

Conceptualization, W.N., X.L., and Y.J.; methodology, W.N., X.L., and Y.J.; investigation, Y.J., X.L., Y.Z., Y.L., Y.G., S.L., C.Z., Y.D., Z.N., L.L., and W.N.; writing – original draft, W.N., X.L., and Y.J.; writing – review & editing, all authors; funding acquisition, W.N.; resources, W.N.; supervision, W.N. and X.L.

## Declaration of interests

The authors declare that they have no known competing financial interests or personal relationships that could have appeared to influence the work reported in this paper.

## STAR★Methods

### Key resources table

**Table:** 

REAGENT or RESOURCE	SOURCE	IDENTIFIER
**Antibodies**		

E-cadherin Monoclonal (ECCD-2)	Invitrogen	Cat#13–1900; RRID: AB_2533005.
γ-tubulin	Sigma-Aldrich	Cat#T5192; RRID: AB_261690.
BrdU	Roche	Cat#11170376001; RRID: N/A.
HRP polymer–goat anti-mouse IgG	Maixin-Bio	Cat#KIT-5010; RRID: N/A.
Alexa Fluor® 594 AffiniPure™ Goat Anti-Rabbit IgG (H + L)	Jackson-Immuno Research	Cat#111-585-144; RRID: AB_2307325.
Goat anti-Rabbit IgG (H + L) Cross-Adsorbed Secondary Antibody, Alexa Fluor™ 488	Invitrogen	Cat#A-11008; RRID: AB_143165.
Biotin-conjugated rabbit anti-rat IgG	Abcam	Cat#Ab6733; RRID: N/A.
Goat Anti-Mouse IgG-FITC	Sigma-Aldrich	Cat#F5262; RRID: N/A.
Sheep Anti-Mouse IgG-Cy3	Sigma-Aldrich	Cat#C2181; RRID: N/A.
Goat anti-Rat IgG (H + L) Cross-Adsorbed Secondary Antibody, Alexa Fluor™ 488	Invitrogen	Cat#A-11006; RRID: AB_2534074.
Mouse FSTL1 antibody	R&D	Cat#AF1738; RRID: AB_354962.
Human FSTL1 antibody	R&D	Cat#AF1694; RRID: AB_2106831.
Smad5	Cell Signaling Technology	Cat#12534 S; RRID: N/A.
Smad1/5/8	Immunoway	Cat#YT4325; RRID: N/A.
*p*-Smad1/5/8	Cell Signaling Technology	Cat#13820; RRID: AB_2493181.
ERK	Cell Signaling Technology	Cat#4695; RRID: N/A.
*p*-ERK	Cell Signaling Technology	Cat#4370 S; RRID: N/A.
BMP4	Proteintech	Cat#12492-1-AP; RRID: AB_2063531.
Wnt5a	R&D	Cat#AF645; RRID: AB_2288488.
Ror2	Santa cruze	Cat#sc-374174; RRID: AB_10989358.
β-actin	ABclonal	Cat#AC026; RRID: AB_2768234.
β-tubulin	Cell Signaling Technology	Cat#2128 L; RRID: N/A.
GAPDH	Utibody Biotech	Cat#UM4002; RRID: AB_3665266.
secondary antibodies FITC	Invitrogen	Cat#11-4317-87; RRID: N/A.
Goat anti-Mouse IgG (H + L)	Thermo	Cat#31430; RRID: AB_228307.
Goat anti-Rabbit IgG (H + L)	Thermo	Cat#31460; RRID: AB_228341.
Goat IgG HRP-conjugated Antibody	R&D	Cat#HAF109; RRID: AB_357236.

**Chemicals, peptides, and recombinant proteins**		

Dorsomorphin	Aladdin	D139352
DMSO	Sigma	276855
Paraformaldehyde	Sigma	P6148
Paraffin	CITOTEST	80200
OCT	Sakura	4583
Amersham Hybond P 0.45 μm PVDF	Cytiva	10600023
Recombinant Noggin	R&D	TNT3121044
FBS	Gibco	10270–106
Penicillin	Yuanye Bio-Tech	S17030
Streptomycin	BBI	A610494-0050
Trypsin	Gibco	25200–072
Sodium citrate buffer	Maixin-Bio	MVS-0100
Collagen Type Ⅰ	Corning	354236
Trizol reagent	Invitrogen	15596018
DAPI	Roche	70473520
Tyramide Reagent	Perkin Elmer	LS-3500
Phosphatase inhibitor	Bimake	B15002
ABC reagent	Vector Laboratories	PK-7100
Recombinant Human BMP4 Protein	R&D	Q53XC5
Color Prestained Protein Marker (10–180 kDa)	GenStar	M221
Color Prestained Protein Marker (10–180 kDa)	Life-iLab	AP13L053
BrdU reagent	Sigma	B5002
3,3-diaminobenzidine substrate	Maixin-Bio	DAB-2031
Triton X-100	Sangon Biotech	9002-93-1
Bovine serum albumin	Sigma	9048-46-8

**Critical commercial assays**		

One Step Mouse Genotyping Kit	Vazyme	PD101-01
Transgene Reverse Transcription Kit	YEASEN	11141ES60
SYBR Green Master Mix	YEASEN	11184ES08
Mouse BMP4 ELISA kit	CUSABIO	CSB-E04512m
BCA Protein Assay Kit	Thermo	23228
10%-separation gel solution	YEASEN	20325-B
Star Signal	GenStar	ZE171-101

**Deposited data**		

Single cell sequencing of E8.0-E9.5 mouse foregut	Han et al.^17^	GEO: GSE136689
Single cell sequencing of E9.5-E11.5 mouse embryonic lungs	Dong et al.^18^	GEO: GSE87038
Single cell sequencing of E12.5 mouse embryonic lungs	Cohen et al.^19^	GEO: GSE119228
Single cell sequencing of E18.5 mouse embryonic lungs	Zhang et al.^15^	GEO: GSE225463

**Experimental models: Cell lines**		

BEAS-2B	ATCC	RRIDs: CVCL_0168
MLE12	ATCC	RRIDs: CVCL_3751

**Experimental models: Organisms/strains**		

*Fstl1*^+/−^ mouse	Geng et al.^11^	N/A

**Oligonucleotides**		

RNAscope Probe- Mm-*Bmp4*-C1	ACD	401301
RNAscope Probe- Mm-*Fstl1*-C1	ACD	534121
Primers for PCR and qRT-PCR, see Table S1	This paper	N/A

**Software and algorithms**		

ImageJ	National Institutes of Health	https://imagej.net/ij/download.html
Cellranger	10× Genomics	https://www.10xgenomics.com/support/software/cell-ranger/downloads
Seurat (version 5.3.0)	Satija Lab	https://cran.r-project.org/src/contrib/Archive/Seurat/
GraphPad Prism 10	GraphPad Software	https://www.graphpad.com/features
Laser Scanning Confocal Microscope	Carl Zeiss	Zeiss LSM 710
Laser Scanning Confocal Microscope	Leica Microsystems	Leica TCS SP5
Light microscope	Olympus	DP73
Line Gene 9600 System	BIOER	FQD-96 A
Chemiluminescence imaging system	JUNYI	MiniChemi 610

### Experimental model and study participant details

#### Animals models

All animals were housed and cared for in a specific-pathogen free (SPF) facility at Nankai University in humidity-controlled and temperature-controlled rooms on a 12 h light-dark cycle with free access to food and water. All animal experimental protocols were approved by the Animal Care and Use Committee of Nankai University (approval no. 20140008).

*Fstl1*-deficient mice (*Fstl1*^*+/−*^)^11^ were previously described. Mice had mixed genetic backgrounds. Noon of the day on which a vaginal plug was detected was considered E0.5. Pregnant mice were sacrificed by cervical dislocation, and the embryos were isolated quickly after uteri were removed and used for subsequent experiments. Both male and female mice were used for the experiments. All efforts were made to minimize animal suffering, and all approaches were used to minimize the number of mice.

#### Administration of inhibitors to mice

The pregnant mice were treated with 5 mg/kg Dorsomorphin (Aladdin) or 5 mg/kg DMSO (Sigma) by intraperitoneal injection each day.

#### Genotyping

The genotypes of mice were determined by polymerase chain reaction (PCR). Genomic DNA was isolated from mouse tails or nails by alkaline lysis method(Vazyme). A 676-bp fragment was produced from the WT allele (*Fstl1*^*+/+*^), while a 350-bp fragment was produced from the null allele (*Fstl1*^−/−^). The primers are listed in Table S1.

#### Cell lines

BEAS-2B cells were purchased from ATCC (RRIDs: CVCL_0168) and maintained in Dulbecco’s modified Eagle medium (DMEM, Gibco) with 10% fetal bovine serum (FBS, Gibco) and antibiotics (penicillin and streptomycin). BEAS-2B was selected as a well-characterized model of human bronchial epithelium, which was critical for our study. Prior to use, the cell line was confirmed to be contamination free. MLE12 cells were purchased from ATCC (RRIDs: CVCL_3751) and maintained in Dulbecco’s modified Eagle medium/F-12 (DMEM/F-12, Gibco) with 10% fetal bovine serum (FBS, Gibco) and antibiotics (penicillin and streptomycin). MLE12 was selected as a well-characterized model of lung epithelium, which was critical for our study. Prior to use, the cell line was confirmed to be contamination free.

#### Mouse embryonic fibroblasts (MEFs) isolation and culture

MEFs were isolated from mice at 13.5 days of embryonic stage (E13.5). In brief, *Fstl1*^*+/−*^ mice were intercrossed to generate *Fstl1*^*+/+*^ and *Fstl1*^−/−^ embryos, and noon of the day on which a vaginal plug was detected was considered E0.5. Pregnant mice at E13.5 were euthanized by cervical dislocation. Embryos were rapidly dissected, and the head, tail, limbs, and internal organs were removed. The remaining tissues were collected for subsequent analyses, which were cut into small pieces and then incubated in 0.05% Trypsin (Gibco) for 30 min at 37°C. DMEM was added to stop enzymatic reaction. After centrifugation at 1600 rpm for 10 min, the cell pellets were resuspended and cultured in DMEM supplemented with 10% FBS and antibiotics in 5% CO2 at 37°C in a humidified atmosphere. Cells of passage 3 were used for subsequent experiments. Both male and female E13.5 embryos were used for the experiments.

### Method details

#### Trachea hematoxylin and eosin (H&E) staining

Embryos were harvested and fixed in 4% paraformaldehyde (Sigma) at 4°C overnight. For paraffin-sections, after fixation, samples were dehydrated with gradient concentration ethanol and embedded in paraffin (CITOTEST) for sectioning, 5 μm sections were cut from paraffin-embedded blocks. For cryosections, after fixation, samples were placed in 30% sucrose (BBI) and embedded in OCT (Sakura), 20 μm sections were cut from OCT-embedded blocks. Transverse sections obtained from the middle location of the trachea were utilized for subsequent experiments. Sections stained with hematoxylin and eosin were analyzed to measure the tracheal lumen circumference and area. Digitized images were analyzed using ImageJ software.

#### Trachea immunofluorescence staining

Immunofluorescence staining was performed as previously described. In brief, 5 μm paraffin-sections were deparaffinized and rehydrated, 20 μm cryosections were immersed in PBS to remove the OCT. ‌Antigen retrieval was performed‌ by high-pressure and high heating with sodium citrate buffer (Maxin-Bio). Sections were incubated with the following primary antibodies overnight at 4°C with indicated dilutions: rabbit anti-*p*-Smad1/5/8 (CST, 13820, 1:100), rabbit anti-*p*-ERK (CST, 4370, 1:1000), rat anti-E-cadherin (Invitrogen, 131900, 1:100), rabbit anti-γ-tubulin (Sigma, T5192, 1:100), Then sections were incubated with Alexa Fluor 488-conjugated secondary antibodies (Invitrogen, A11006, 1:200) and Alexa Fluor 594-conjugated secondary antibodies (Jackson Immuno Research, 111-585-144, 1:200), FITC secondary antibodies (Sigma, F5262, 1:200) and Cy3 secondary antibodies (Sigma, C2181, 1:200). Nucleus were labeled with 25 μg/mL DAPI (Roche). For the immunofluorescence staining of *p*-Smad1/5/8 and *p*-ERK, 1X phosphatase inhibitor was added in 4% PFA during the tissue fixation process. The tyramide signal amplification (PerkinElmer) and the ABC method (Vector Laboratories) were used for *p*-Smad1/5/8 and *p*-ERK staining followed the manufacturer’s recommendations. Images were captured using TCS SP5 confocal microscope (Leica) and LSM710 confocal microscope (Zeiss). The circumference of tracheal lumen and the numbers of tracheal epithelial cells that located within the apical surface were measured, mean length of apical cells in tracheal epithelium was analyzed to investigate the rearrangement of tracheal epithelial cells.

#### BrdU immunohistochemistry (IHC)

100 mg/kg BrdU (Sigma) was injected intraperitoneally 4 h prior to sacrificing the pregnant mice, embryos from pregnant mice were harvested and fixed in 4% paraformaldehyde. Paraffin-section slides were treated with 1 N HCl for 1 h at 37°C, 0.1 M Borate was used to neutralize the acid, then sections were incubated with BrdU antibody (Roche)‌ at 4 °C. HRP polymer–goat anti-mouse IgG (Maixin-Bio) was incubated for 15 min at room temperature. Finally, signals were detected by using 3,3-diaminobenzidine substrate (Maixin-Bio). Images were captured using light microscope (Olympus).

#### Fluorescent whole-mount antibody staining with anti-E-cadherin

Embryos from pregnant mice were harvested and fixed in 4% paraformaldehyde, then, the specimens were washed in MeOH and 5% H_2_O_2_. E-cadherin antibody (Invitrogen, 131900, 1:100) was incubated overnight at 4 °C, biotin-conjugated rabbit anti-rat IgG (Abcam, Ab6733, 1:500) was incubated overnight at 4 °C, specimens were incubated with ABC Elite reagent (Vector Laboratories) for 2 h at room temperature, finally specimens were incubated with Tyramide Reagent (PerkinElmer). Images were captured using TCS SP5 confocal microscope (Leica) and LSM710 confocal microscope (Zeiss).

#### Determination of mitotic spindle angles in tracheal epithelial cells

To determine the mitotic spindle angles of tracheal epithelial cells, 20 μm cryosections of trachea at given embryonic stages were stained with antibody against E-cadherin to label the epithelium, γ-tubulin to label the mitotic spindle centrosome and DAPI to label the DNA. Z-stacks of the mitotic spindle poles in each dividing cell at anaphase and telophase were determined by confocal microscope (Leica and Zeiss) and the distance parallel to the tracheal longitudinal axis (ΔZ stack) was determined by the two Z-stacks. The confocal images at the two Z-stacks were then merged and the distance perpendicular to the tracheal longitudinal axis (ΔX-Y) were calculated by matching the distances of mitotic spindle poles to scale bar in ImageJ. The mitotic angles (θ) of the epithelial cells were determined by θ = ATAN(ΔX-Y/ΔZ) ∗180/3.14.

#### BEAS-2B and MEFs co-culture system

BEAS-2B cells were cultured in the Collagen I (Corning) coated upper chamber of a transwell with 0.4 μm pore size (NEST), whereas primary MEFs cells of passage 3 were cultured in the lower plate. BMP4 inhibitors 25 ng/mL Noggin (R&D) was added separately in the lower plate medium since Day 0 of culture. After 1 h of Noggin treatment, BEAS-2B cells were harvested to analyze the expression of *p*-Smad1/5/8. After 2 days culture, the upper chamber was collected for cell immunofluorescence staining to determine the mitotic spindle angles of BEAS-2B cells and was collected for qRT-PCR to determine the expression of *ID1*, *ID2* and *ID3*.

#### BMP4 treatment of BEAS-2B and MLE12 cells

BEAS-2B cells and MLE12 cells were stimulated with 20 ng/mL BMP4 (R&D). After 1 h of BMP4 treatment, BEAS-2B cells were harvested for western blot analysis to assess the expression of *p*-Smad1/5/8 and *p*-ERK. Two days after stimulation, BEAS-2B and MLE12 cells were collected and processed for immunofluorescence staining to determine the mitotic spindle angles.

#### Cell immunofluorescence staining

Cells grown on culture dish were fixed in 4% paraformaldehyde for 20 min at room temperature, permeabilized with 0.2% Triton X-100 for 30 min, 5% bovine serum albumin was added to block non-specific protein for 1 h. The following primary antibody was incubated overnight at 4°C with indicated dilutions: rabbit anti-γ-tubulin (Sigma, T5192, 1:100). Alexa-Fluor-594-conjugated secondary antibody (Jackson Immuno Research, 111-585-144, 1:200) was incubated at room temperature for 2 h. After antibody staining, nuclei were stained with 25 μg/mL DAPI (Roche) at room temperature for 1 h.

#### Determination of mitotic spindle angles in BEAS-2B and MLE12 cells

To determine the mitotic spindle angles of BEAS-2B and MLE12 cells *in vitro*, cells were stained to immunofluorescence staining for γ-tubulin to label the spindle poles. The mitotic angle (α) of epithelial cells was then calculated based on the vertical (ΔZ) and horizontal (X) distances between mitotic spindle poles, using the formula α = ATAN(ΔZ/X) × 180/π.

#### RNA extraction and qRT-PCR

Total RNA was extracted using Trizol reagent (Invitrogen) according to the manufacturer’s instructions, cDNA was prepared using a Transgene Reverse Transcription Kit (YEASEN), qPCR mixtures were prepared using the SYBR Green Master Mix (YEASEN) and sequences were amplified with a Line Gene 9600 System (BIOER, China). Expression of *Fstl1* and *Bmp*4 were determined. Mouse *β-actin* was used as an internal control. Expression of *ID1*, *ID2* and *ID3* were determined. *GAPDH* was used as an internal control. The sequences of specific primers are listed in Table S1.

#### Western blot

Protein extracts were collected from E16.5 trachea, E18.5 trachea, MEFs and cellular supernatants. Lysates were sonicated and centrifuged. Supernatants were transferred to fresh tubes. Protein concentrations were measured with the application of BCA Protein Assay Kit (Thermo). Equal amounts of protein were separated by 10%-separation gel solution (YEASEN) and transferred to 0.45 μm Polyvinylidene fluoride (PVDF, Cytiva) membranes. A pre-stained protein ladder (GenStar and Life-iLab) was run in parallel to confirm the molecular weight of the target proteins. The membranes were blocked with 5% Nonfat-Dried milk (Coolaber) in TBST for 1 h at room temperature and incubated with primary antibody overnight at 4 °C. Following primary antibodies were used to recognize proteins: Smad1/5/8 (Immunoway, YT4325, 1:1000), *p*-Smad1/5/8 (CST, 13820, 1:1000), FSTL1 (R&D, AF1738, 1:1000), ERK (CST, 4695, 1:1000), *p*-ERK (CST, 4370, 1:1000), Wnt5a (R&D, AF645, 1:1000), Ror2 (Santa cruze, sc-374174, 1:1000), BMP4 (Proteintech, 12492-1-AP, 1:1000), β-actin (Abclonal, AC026, 1:5000), β-tubulin (CST, 2128 L, 1:5000), GAPDH (Utibody Biotech, 1:5000). Next day, the membranes were incubated with the appropriate horseradish peroxidase (HRP)-conjugated secondary antibodies for 2 h at room temperature. Following secondary antibodies were used: HRP-conjugated anti-Rabbit (Thermo, 31460, 1:5000), HRP-conjugated anti-Mouse (Thermo, 31430, 1:5000), HRP-conjugated anti-Goat (Santa cruz, sc-2020, 1:5000). Immunoreactivity was detected by Star Signal (GenStar). Digital images were taken with X-ray film processing and a chemiluminescence imaging system (MiniChemi 610).

#### ELISA

Levels of BMP4 proteins in *Fstl1*^*+/+*^ and *Fstl1*^−/−^ MEFs cellular supernatants were measured with commercial ELISA BMP4 kits (CUSABIO) according to the manufacturers’ instructions.

#### RNAscope *in situ* hybridization

Tissues were harvested, fixed in 4% paraformaldehyde for 4 h at room temperature, cryoprotected in 30% sucrose (BBI) overnight at 4°C, and then embedded in OCT. Tissues sections were cut at 10 μm thickness, air-dried at room temperature and processed for RNA *in situ* detection by using RNAscope 2.0 kit according to the manufacturer’s instructions (ACD). We used the following RNAscope probe: *Bmp4* (401301), *Fstl1* (534121).

#### Expression profile dataset and analysis

Three recently published single cell RNA-seq datasets in the National Center of Biotechnology Information Gene Expression Omnibus under accession no. GSE136689, GSE87038 and GSE119228 were acquired and analyzed. Seurat was used to remove poor quality cells. Gene counts were normalized using the Normalize Data method and 3000 highly variable genes were selected for downstream analysis. Canonical Correlation Analysis (CCA) method was used for dataset integration to correct batch effects, anchors were determined using the Find Integration Anchors function, then Integrate Data was used to generate an integrated assay. RunPCA was applied for dimensional reduction. Cells were clustered using Seurat and visualized using UMAP. A resolution of 0.5 was chosen to initialize the clusters. The canonical markers *Epcam*, *Foxd1* were used to identify epithelial cell and mesenchymal cell populations. The expression of genes was visualized using Violin plot in Seurat.

We previously published scRNA-seq data from *Fstl1*^*+/+*^ and *Fstl1*^−/−^ lungs accession no. GSE225463. A resolution of 0.8 was chosen to initialize the clusters. We isolated proximal airway epithelial cells (Epcam^+^Sox2^+^) and assessed the expression of *Id1*, *Id2* and *Id3*.

### Quantification and statistical analysis

All experiments were repeated at least three times independently with similar results. Data are expressed as the mean ± SEM. Differences between groups were assessed by using Student’s *t*-tests or one-way ANOVA. Results were considered statistically significant at *p* < 0.05. ∗, *p* < 0.05; ∗∗, *p* < 0.01; ∗∗∗, *p* < 0.001; ∗∗∗∗, *p* < 0.0001. GraphPad Prism 10 software was used for statistical analysis.
