# Supplementary material for: Fstl1 determines luminal size of mouse trachea through negative regulation of BMP4 signaling
Source: iScience. 2026 Jul 30;29(8):116972. doi: 10.1016/j.isci.2026.116972 (PMC13452180; doi:10.1016/j.isci.2026.116972)

iScience, Volume *29*

Supplemental information

Fstl1 determines luminal size of mouse trachea through negative regulation of BMP4 signaling

Yueyue Jin, Yue Zhang, Yingying Liu, Maolin Yao, Chenjie Zhang, Shuzi Li, Yibo Ding, Zhuan Niu, Yue Han, Yan Geng, Lian Li, Xue Liu, and Wen Ning

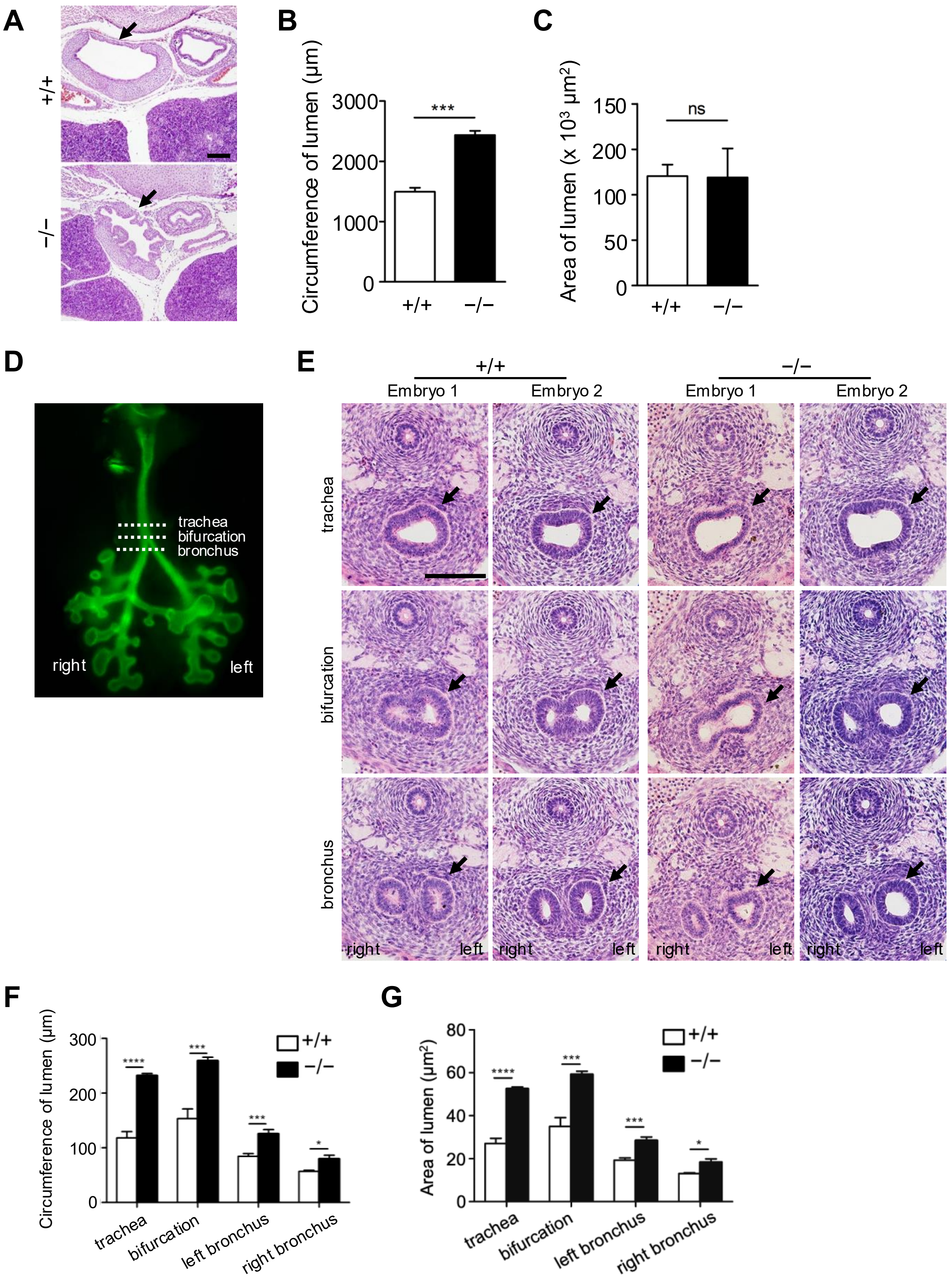

Figure S1. Enlarged tracheal lumen in *Fstl1* deficient mouse embryos at P0, enlarged trachea and main bronchi lumens in *Fstl1* deficient embryos at E12.5.

(A) Representative H&E staining images of $Fstl1^{+/+}$ and $Fstl1^{-/-}$ mouse tracheas at P0. Scale bar: 100 µm.

(B-C) Quantification of circumference (B) and area (C) of $Fstl1^{+/+}$ and $Fstl1^{-/-}$ mouse tracheal lumen at P0. n = 4 each pair. ***, $p < 0.001$, ns, not significant.

(D-E) Whole-mount staining (D) and representative H&E staining images of $Fstl1^{+/+}$ and $Fstl1^{-/-}$ embryonic trachea and main bronchi at E12.5 (E). Scale bar: 100 µm.

(F-G) Quantification of the circumference (F) and area (G) of the $Fstl1^{+/+}$ and $Fstl1^{-/-}$ tracheas, bifurcation sites, left and right bronchi at E12.5. n = 4 each pair. *, $p < 0.05$, ***, $p < 0.001$, ****, $p < 0.0001$.

Data are expressed as the mean ± SEM. Differences between groups were assessed by using Student's *t*-tests.

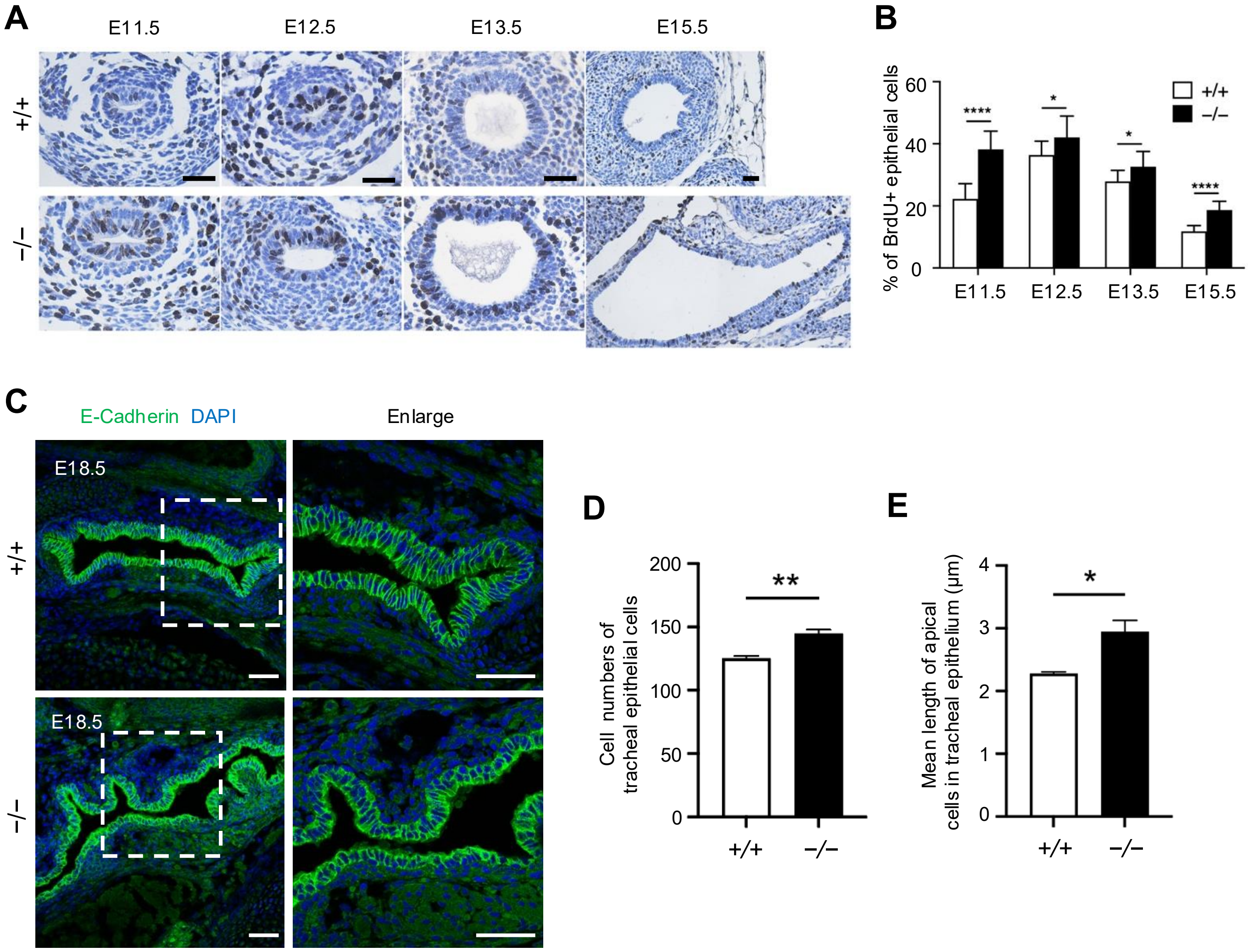

Figure S2. Increased proliferation of tracheal epithelial cells in *Fstl1* deficient mouse.

(A-B) Representative images of immunostaining for BrdU (A) and percentage of BrdU^{+} cells in *Fstl1*$^{+/+}$ and *Fstl1*$^{-/-}$ tracheal epithelial cells in E11.5, E12.5, E13.5 and E15.5 embryos (B). n = 10 per group. *, *$p < 0.05$*, ****, *$p < 0.0001$*. Scale bar: 25 µm.

(C) Immunostaining for E-cadherin on tracheal transverse sections of *Fstl1*$^{+/+}$ and *Fstl1*$^{-/-}$ tracheas at E18.5. Scale bar: 20 µm.

(D) Quantification of the cell numbers of tracheal epithelial cells of *Fstl1*$^{+/+}$ and *Fstl1*$^{-/-}$ tracheas at E18.5. n = 4 each pair. **, *$p < 0.01$*.

(E) Mean length of apical cells in tracheal epithelium of *Fstl1*$^{+/+}$ and *Fstl1*$^{-/-}$ tracheas at E18.5. n = 4 each pair. *, *$p < 0.05$*.

Data are expressed as the mean ± SEM. Differences between groups were assessed by using Student's *t*-tests.

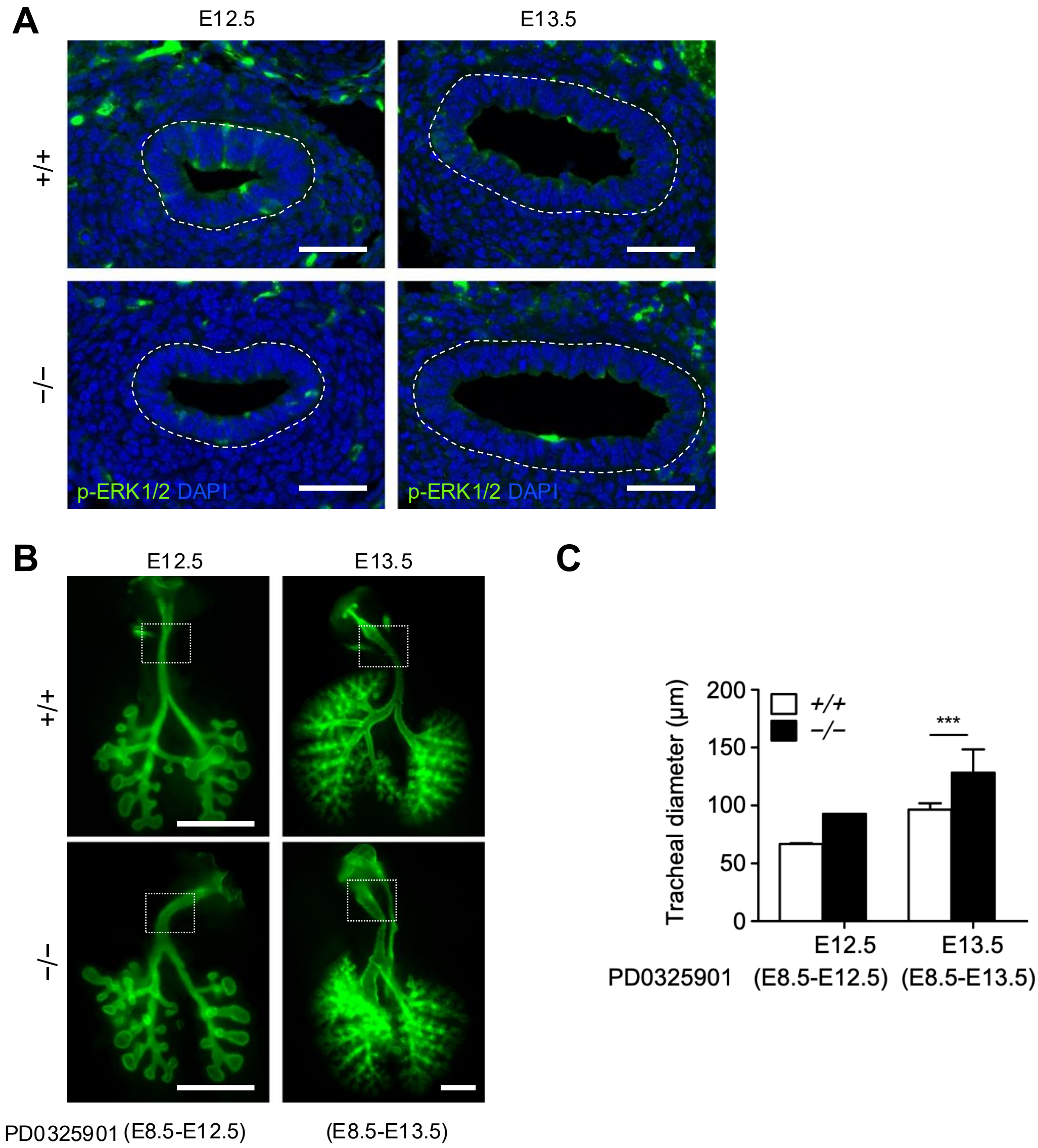

Figure S3. Comparable ERK signaling in *Fstl1*$^{+/+}$ and *Fstl1*$^{-/-}$ tracheal epithelial cells in E12.5 and E13.5 embryos.

(A) Immunostaining of p-ERK1/2 was performed to determine the activity of ERK signaling in E12.5 and E13.5 tracheal epithelial cells. Scale bar, 50 μm.

(B) Whole-mount staining with E-cadherin after the treatment of PD0325901 in E12.5 and E13.5. Scale bar, 500 μm.

(C) Quantification of tracheal diameters after the treatment of PD0325901 in E12.5 and E13.5 embryos. ***, $p < 0.001$.

Data are expressed as the mean ± SEM. Differences between groups were assessed by using Student's *t*-tests.

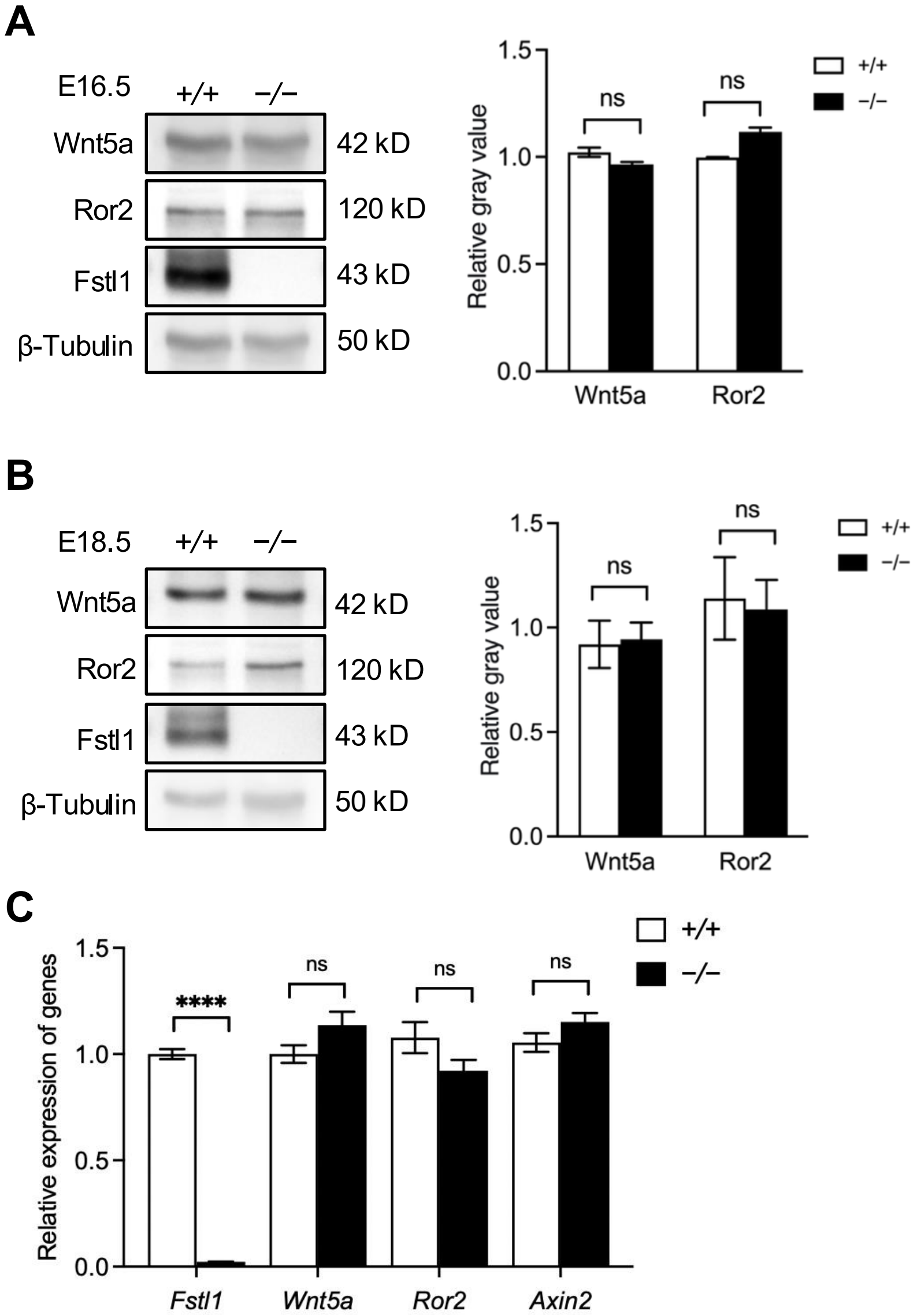

Figure S4. Comparable Wnt signaling in *Fstl1*$^{+/+}$ and *Fstl1*$^{-/-}$ tracheas at E16.5 and E18.5.

(A) Western blot analysis of Wnt5a, Ror2, Fstl1 and β-Tubulin from *Fstl1*$^{+/+}$ and *Fstl1*$^{-/-}$ tracheas in E16.5. Data are representative of two independent experiments.

(B) Western blot analysis of Wnt5a, Ror2, Fstl1 and β-Tubulin from *Fstl1*$^{+/+}$ and *Fstl1*$^{-/-}$ tracheas in E18.5. Data are representative of two independent experiments.

(C) Relative expression levels of *Fstl1*, *Wnt5a*, *Ror2* and *Axin2* in *Fstl1*$^{+/+}$ and *Fstl1*$^{-/-}$ tracheas at E18.5 were determined. n = 9.

Data are expressed as the mean ± SEM. Differences between groups were assessed by using Student's *t*-tests.

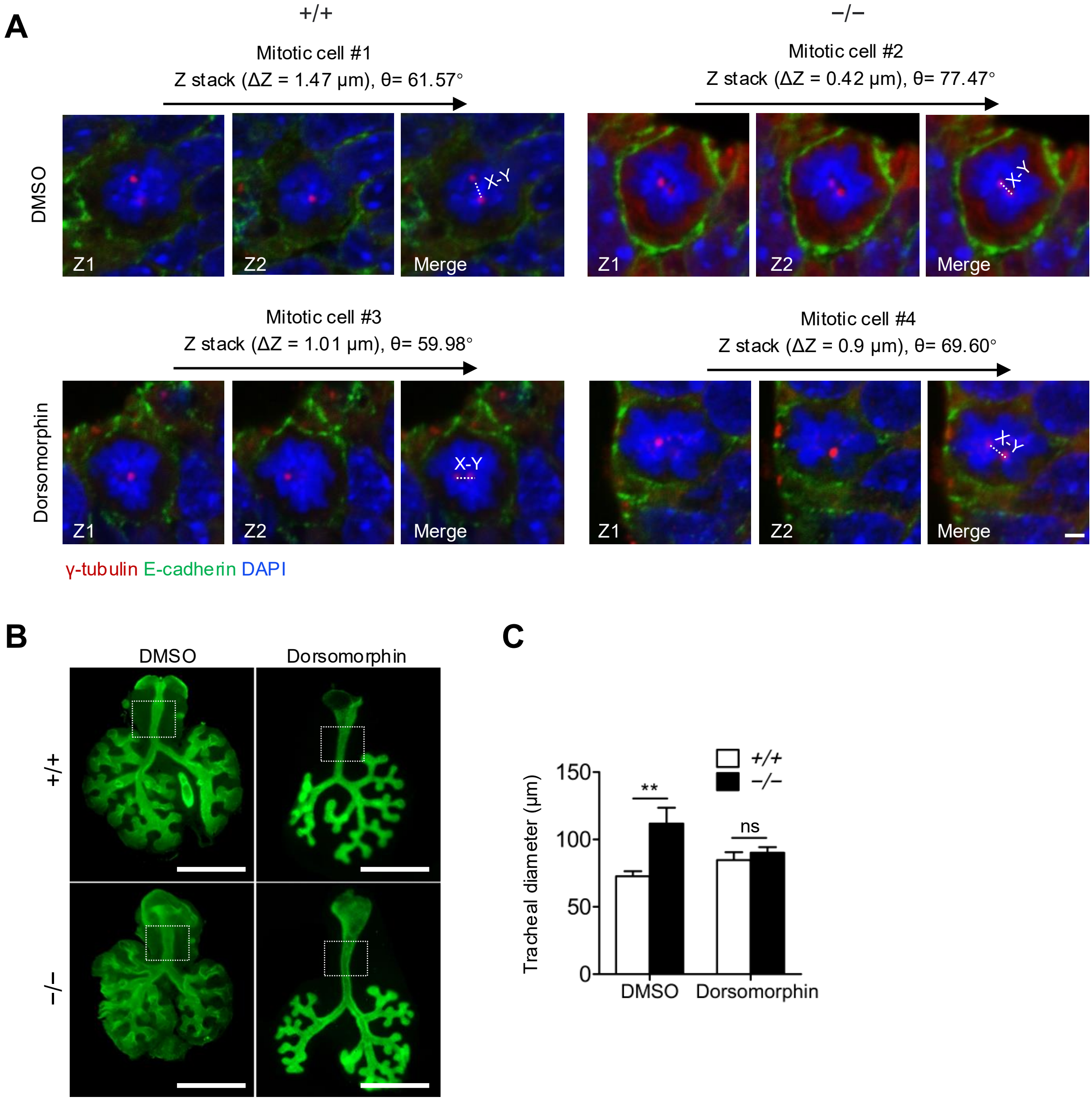

Figure S5. Blocking BMP4 signaling pathway rescued the increased mitotic angles and enlarged tracheal lumen of *Fstl1*-deficient tracheas in E12.5 embryos *in vitro*.

(A) Representative images of mitotic angles in tracheal epithelial cells from embryos treated with Dorsomorphin or DMSO. Immunostaining for centrosomes (γ-tubulin), epithelial cells (E-cadherin), and nuclei (DAPI) were performed to determine the mitotic spindle angles of the tracheal epithelial cells. Scale bar, 2 μm.

(B) Representative images of whole-mount staining with E-cadherin in E12.5 embryos after 48h of *ex vivo* with the treatment of Dorsomorphin. Scale bar, 500 μm.

(C) Quantification of tracheal diameters of E12.5 embryos after 48h of *ex vivo* with the treatment of Dorsomorphin. DMSO, *Fstl1*$^{+/+}$, n = 7, *Fstl1*$^{-/-}$, n = 4; Dorsomorphin, *Fstl1*$^{+/+}$, n = 5, *Fstl1*$^{-/-}$, n = 4; **, $p < 0.01$.

Data are expressed as the mean ± SEM. Differences between groups were assessed by using Student's *t*-tests.

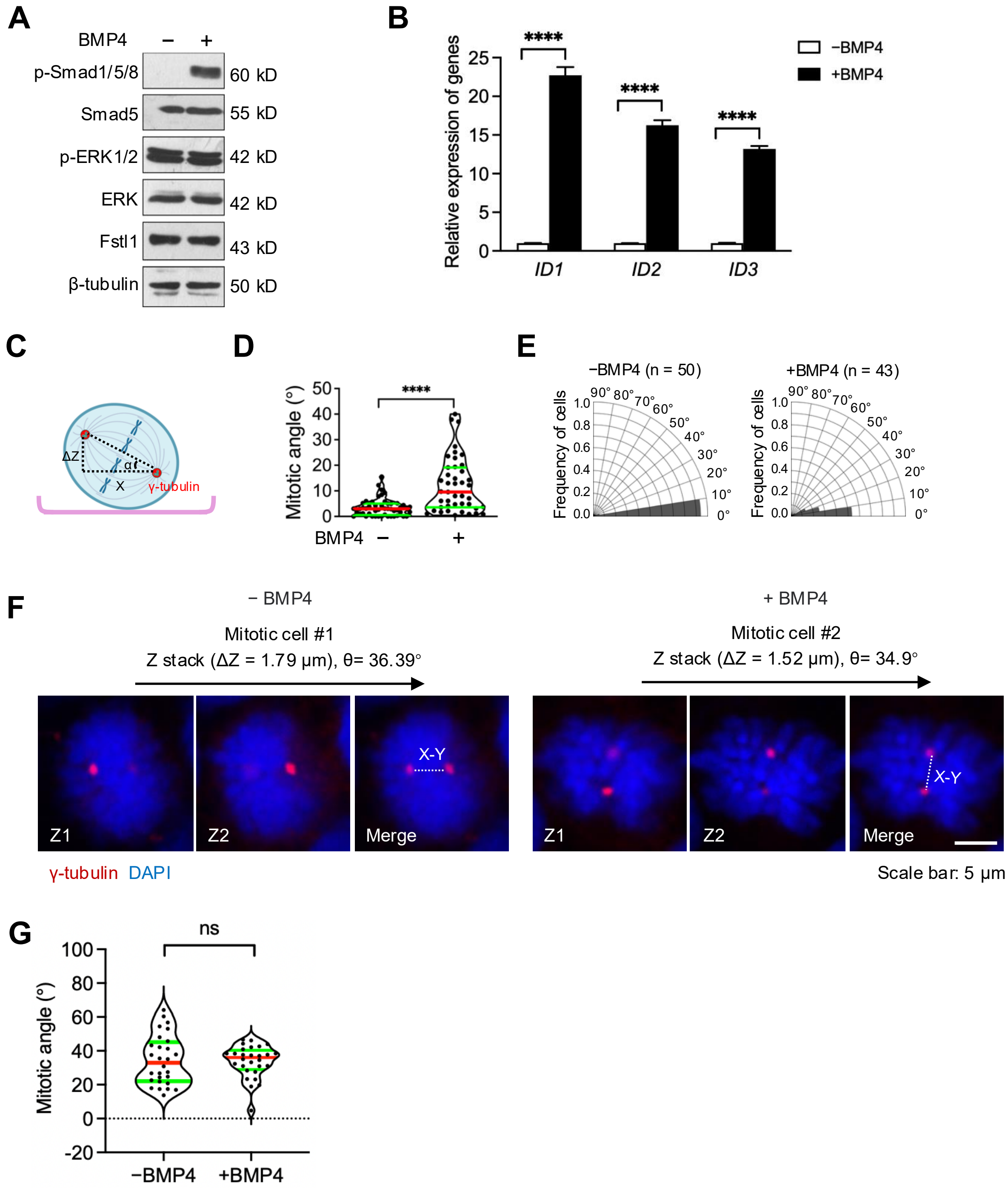

Figure S6. BMP4 regulated the mitotic spindle orientation of BEAS-2B cells.

(A) Western blot analysis of p-ERK1/2 and p-Smad1/5/8 protein levels in BEAS-2B cells with or without the treatment of BMP4 (20 ng/mL). Data are representative of three independent experiments.

(B) Relative expression levels of *ID1*, *ID2* and *ID3* in BEAS-2B cells with or without the treatment of BMP4 (20 ng/mL) were determined by qRT-PCR. n = 3. ****, $p < 0.0001$.

(C) Schematic graph of the mitotic spindle angles of BEAS-2B cells.

(D-E) Violin plots (D) and Rose plots (E) showed the mitotic spindle angles of BEAS-2B cells with or without the treatment of BMP4 (20 ng/mL). ****, $p < 0.0001$.

(F) Representative images showed the mitotic angles of MLE12 cells with or without the treatment of BMP4 (20 ng/mL). Immunostaining for centrosomes (γ-tubulin) and nuclei (DAPI) were performed to determine the mitotic spindle angles of the MLE12 cells. Scale bar, 5 μm.

(G) Violin plots of mitotic spindle angles of MLE12 cells with or without the treatment of BMP4 (20 ng/mL). −BMP4, n = 29, +BMP4, n = 28.

Data are expressed as the mean ± SEM. Differences between groups were assessed by using Student's *t*-tests.

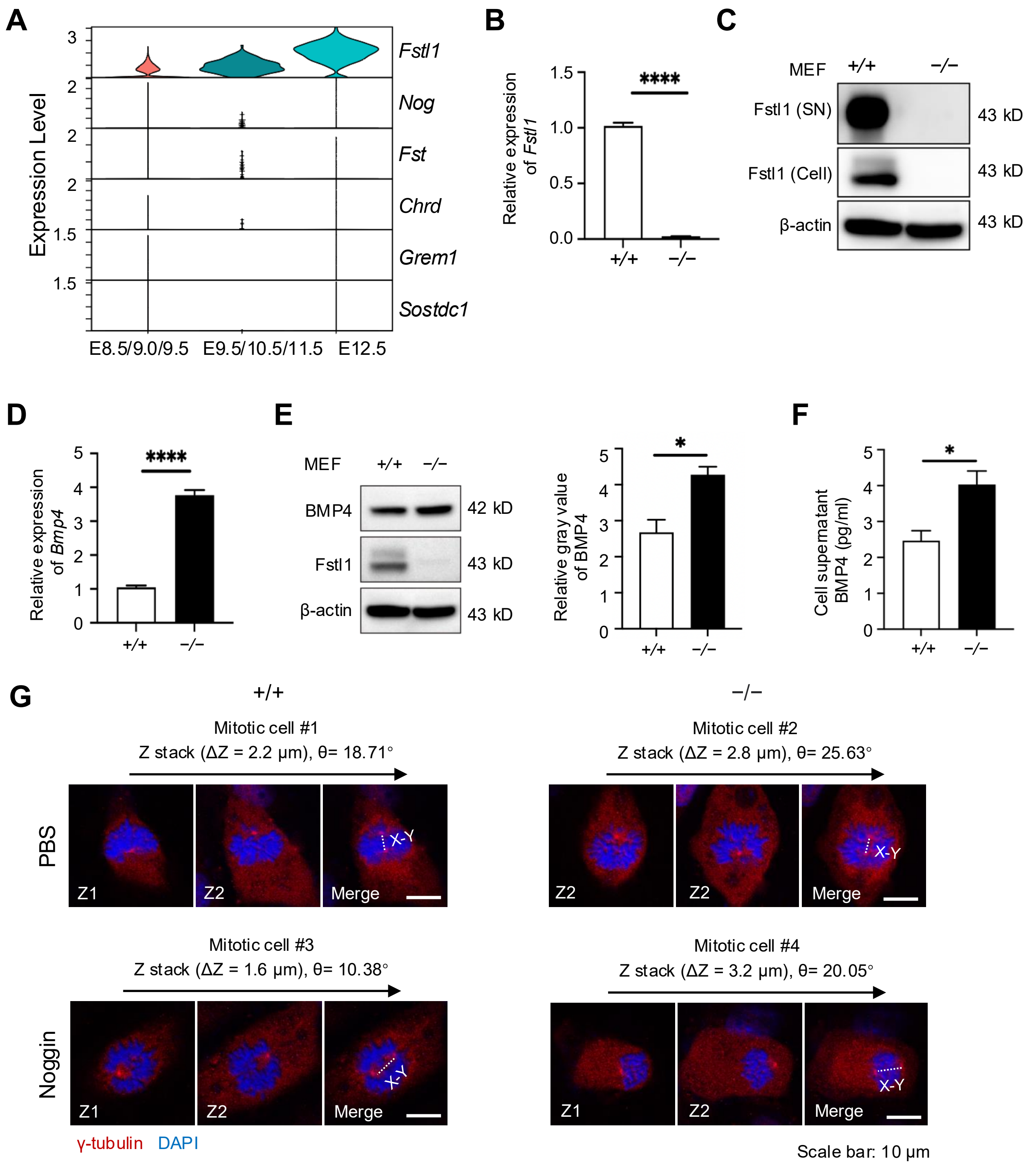

Figure S7. Mesenchyme-derived Fstl1 regulates the mitotic spindle orientation of tracheal epithelial cells through modulation of Bmp4.

(A) Violin plot showed the expression of Bmp4 inhibitor genes in mesoderm of published scRNA-seq data on mouse E8.5/9.0/9.5 foregut (GSE136689), E9.5/10.5/11.5 (GSE87038), and E12.5 (GSE119228) lungs.

(B-C) qRT-PCR (B, n = 3. ****, $p < 0.0001$) and Western blot (C) were used to determined the expression of Fstl1 in MEFs isolated from *Fstl1*$^{+/+}$ and *Fstl1*$^{-/-}$ embryos. Data are representative of three independent experiments.

(D-F) Expression of Bmp4 in MEFs at RNA, total protein, and secretion levels were determined by qRT-PCR (D, n = 3, ****, $p < 0.0001$), Western blot (E, *, $p < 0.05$), and ELISA (F, n = 4, *, $p < 0.05$) analysis, respectively.

(G) Representative images showed the mitotic angles of BEAS-2B cells, co-cultured with MEFs and treated with or without Noggin. Immunostaining for centrosomes (γ-tubulin) and nuclei (DAPI) were performed to determine the mitotic spindle angles of the BEAS-2B cells. Scale bar, 10 μm.

Data are expressed as the mean ± SEM. Differences between groups were assessed by using Student's *t*-tests.

Supplementary Table

Table S1. Primers for PCR and qRT-PCR

	Genes	Forward	Reverse
Primers for PCR	*Fstl1*	GGT CGG ACT TCC TCG GAG CCT GGTG	CCC CCC CGG CTA GGA AAG ACT TGG
Primers for qRT-PCR	*Actin*	AGGCCAACCGTGAAAAGATG	AGAGCATAGCCCTCGTAGATGG
	Fstl1	TTATGATGGGCACTGCAAAGAA	ACTGCCTTTAGAGAACCAGCC
	Bmp4	CCAAACGTAGTCCCAAGCAT	TCAGTTCAGTGGGGACACAA
	GAPDH	GGAGCGAGATCCCTCCAAAAT	GGCTGTTGTCATACTTCTCATGG
	ID1	CTCCTGCGTAGGATCTGAGG	CACCCCCAAGCATACTCCTA
	ID2	TCAGCACTTAAAAGATTCCGTG	GA CAGCAAAGCACTGTGTGG
	ID3	CAGCTTAGCCAGGTGGAAATCC	GTCGTTGGAGATGACAAGTTCCG

Western Blotting images (Data S1)

Figure 3C: Western blot of E16.5 trachea

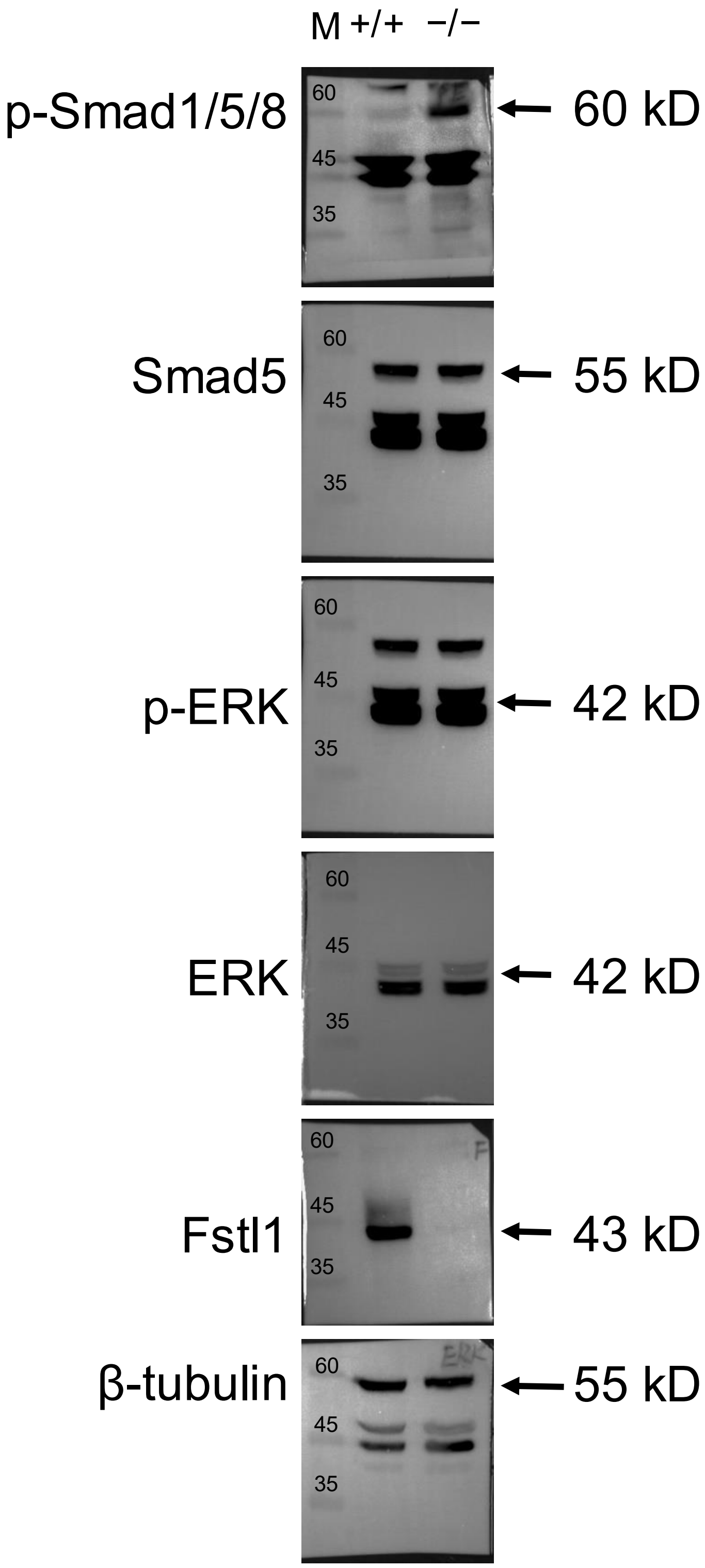

The first independent experiment.

Figure 3C: Western blot of E16.5 trachea

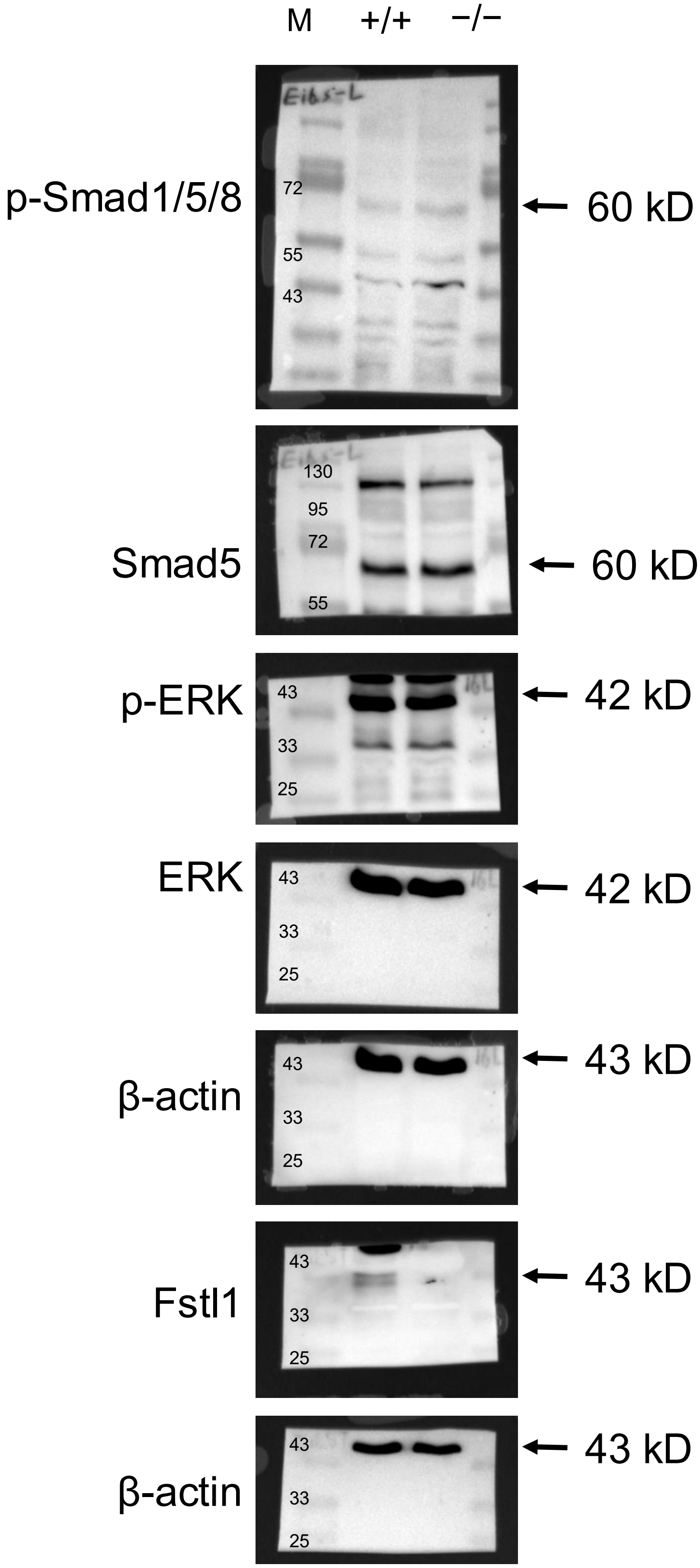

The second independent experiment.

Figure 3D: Western blot of E18.5 trachea

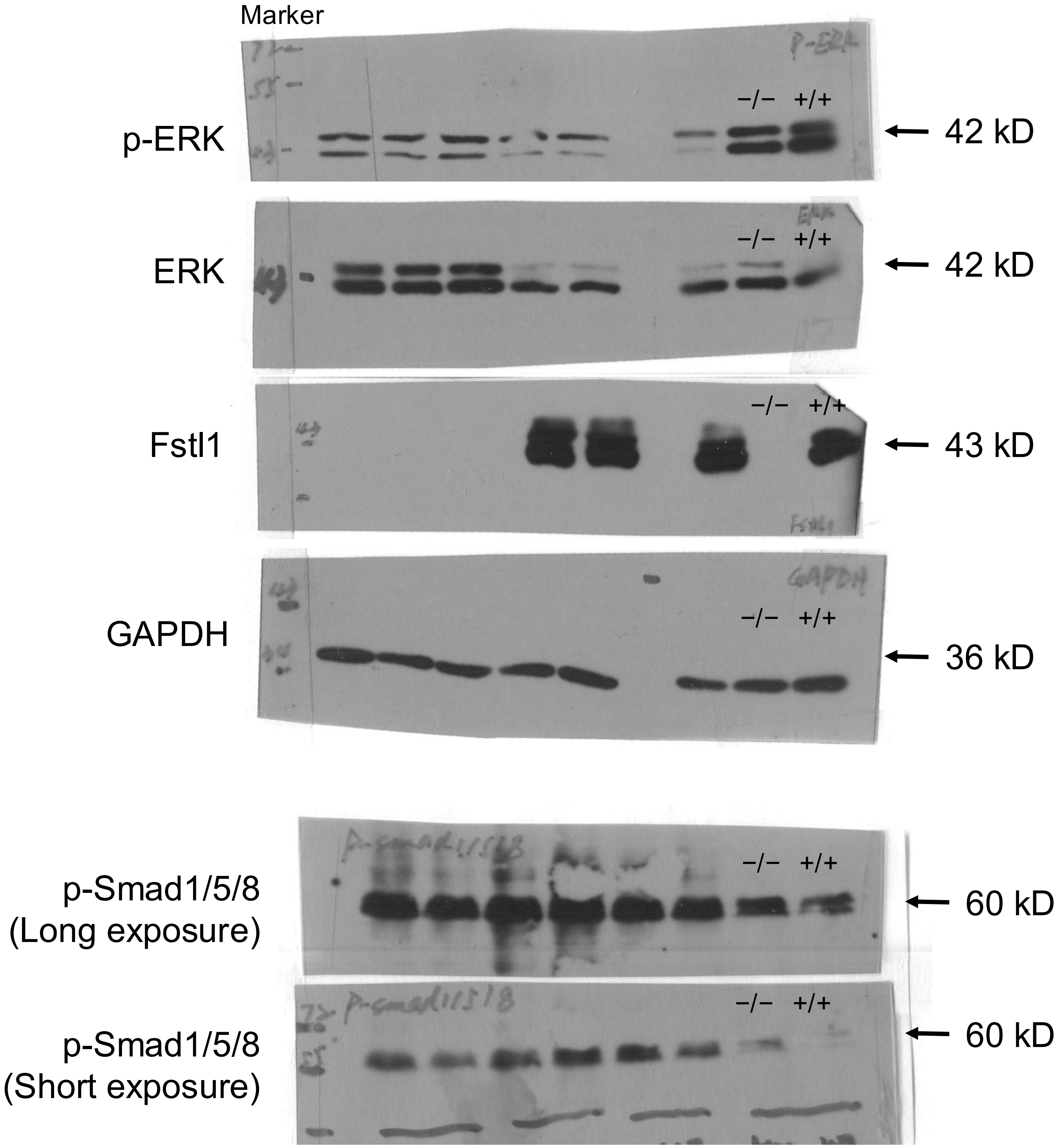

The first independent experiment.

Figure 3D: Western blot of E18.5 trachea

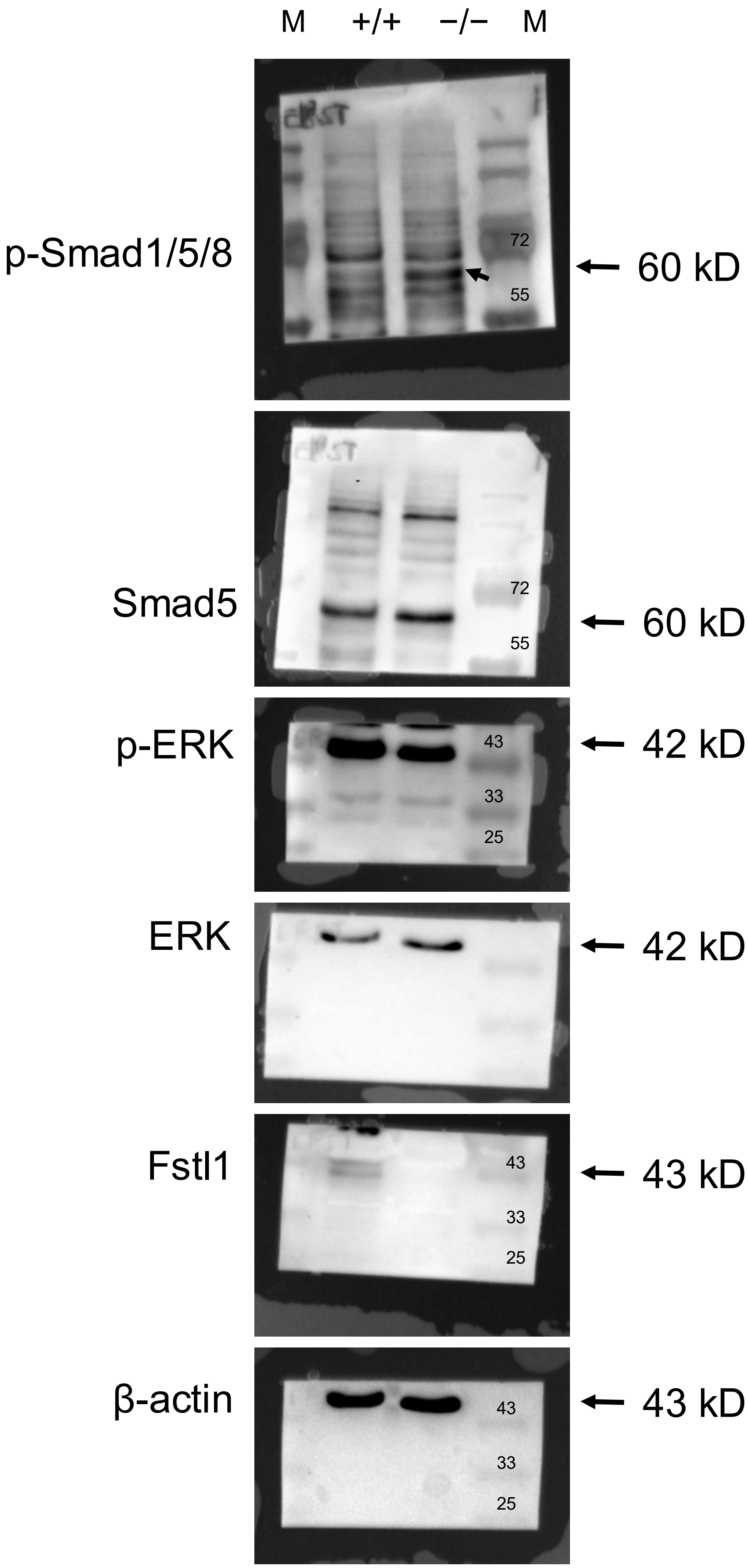

The second independent experiment.

Figure 3D: E18.5 trachea

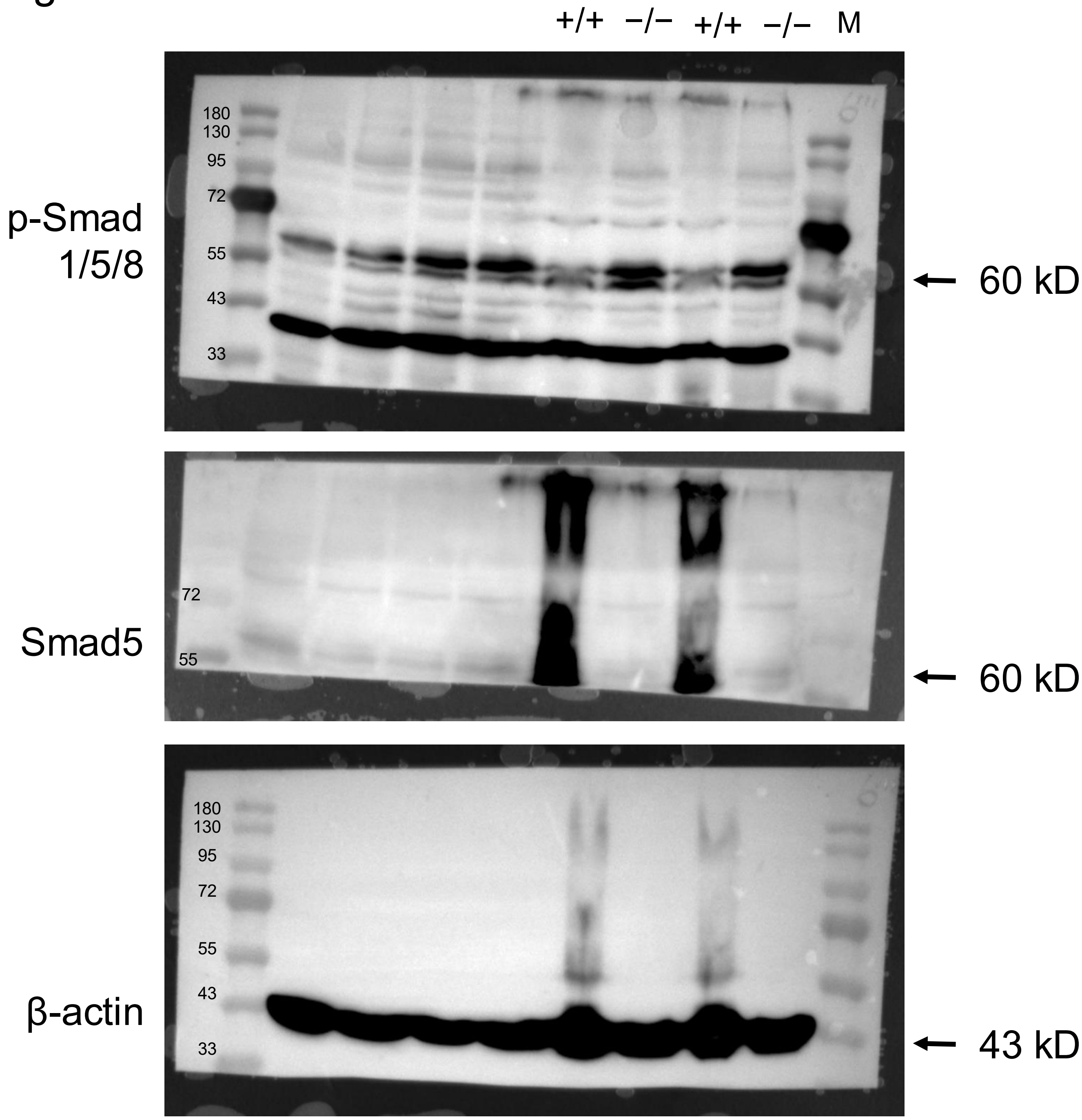

The third independent experiment.

Figure 6B

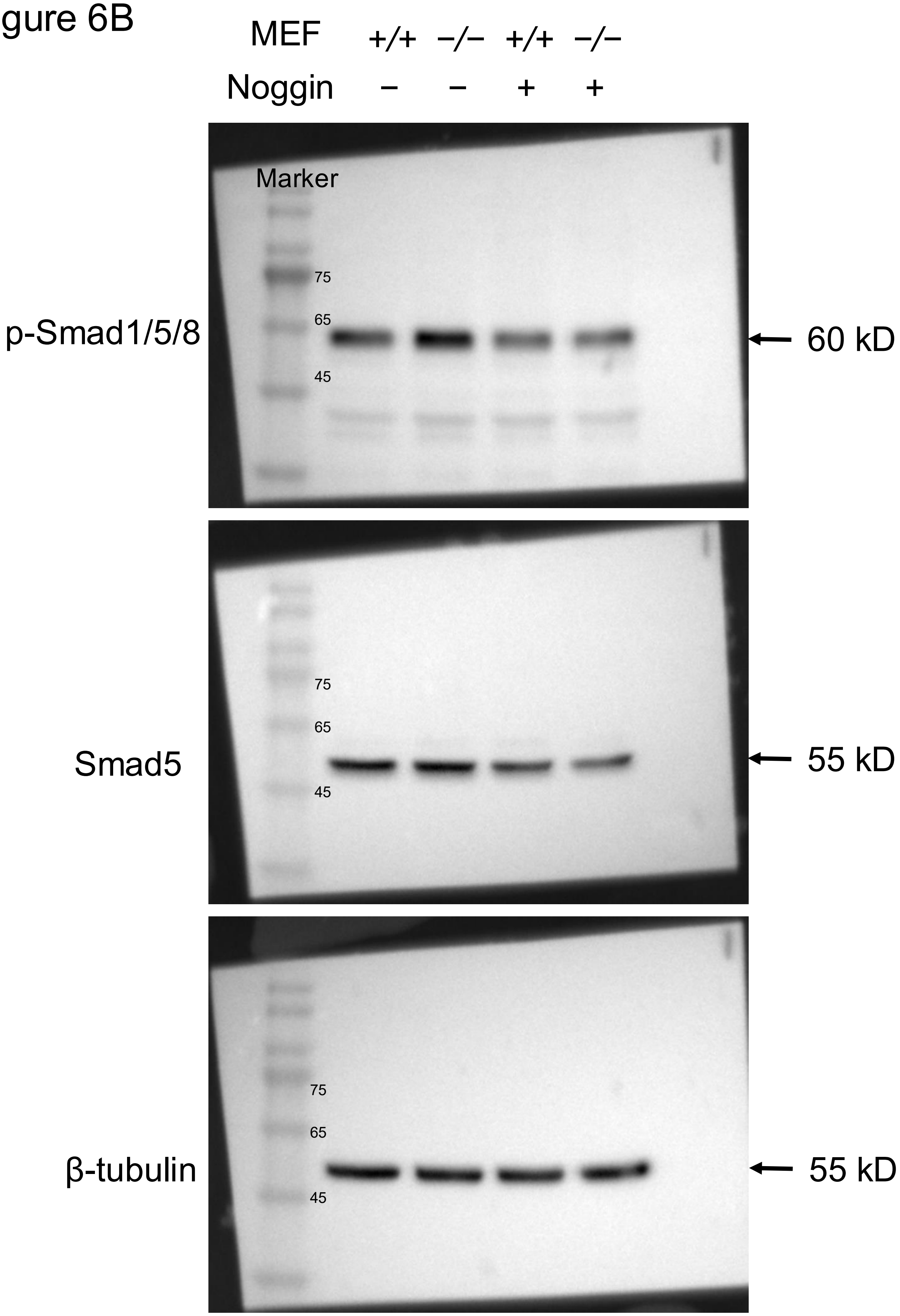

The first independent experiment.

Figure 6B

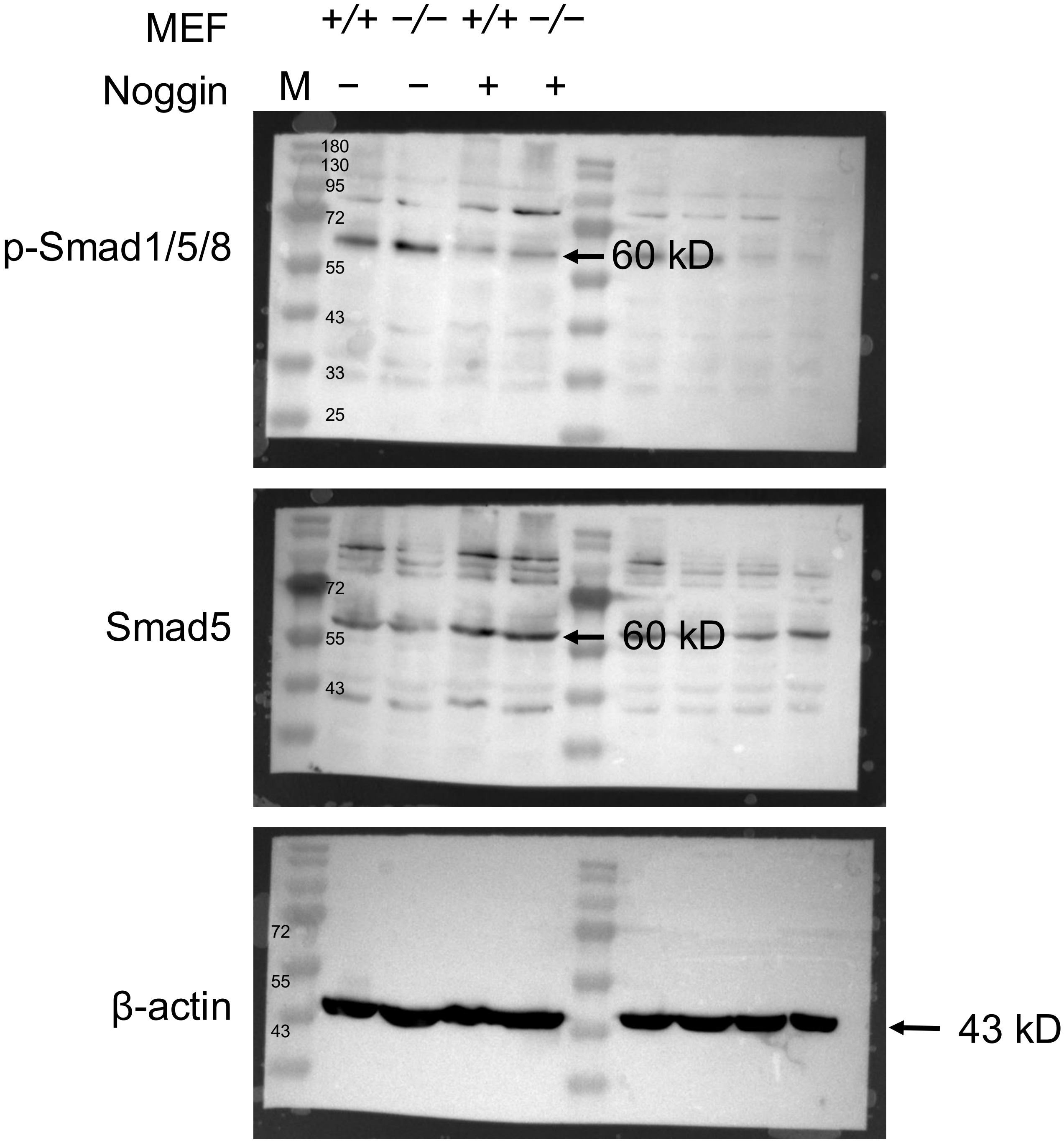

The second independent experiment.

Figure 6B

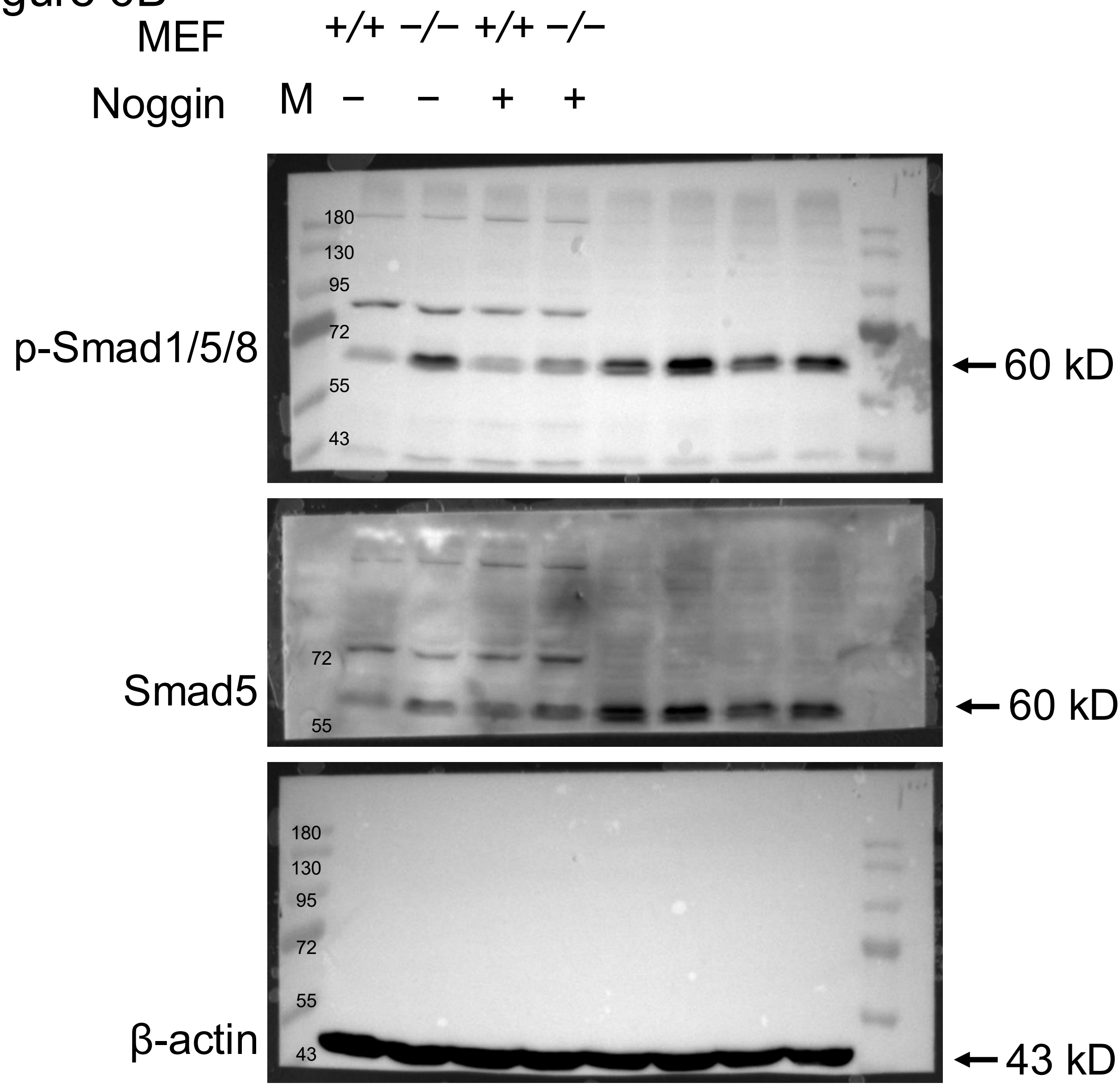

The third independent experiment.

Figure S4A and S4B

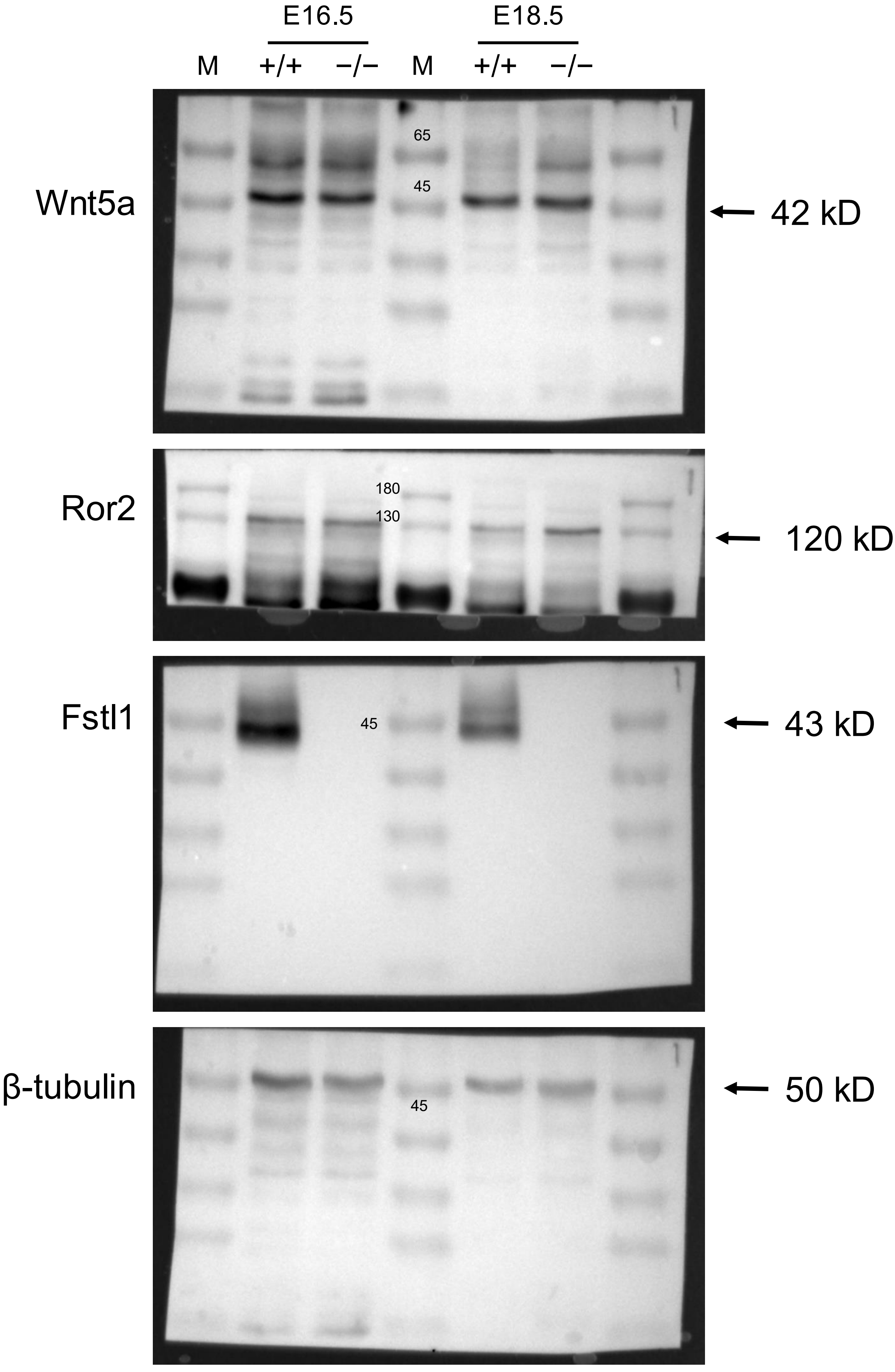

The first independent experiment.

Figure S4A and S4B

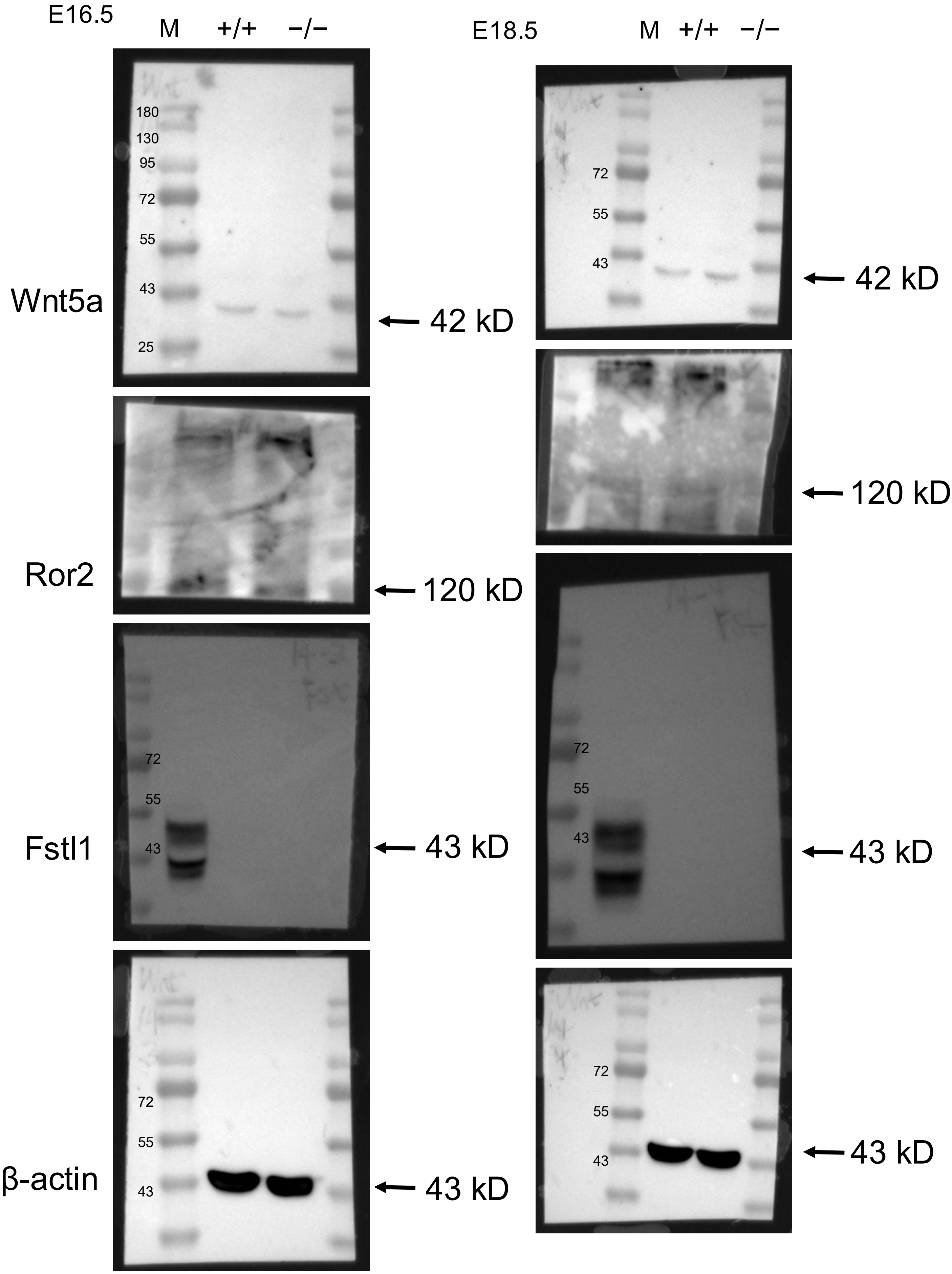

The second independent experiment.

Figure S6A

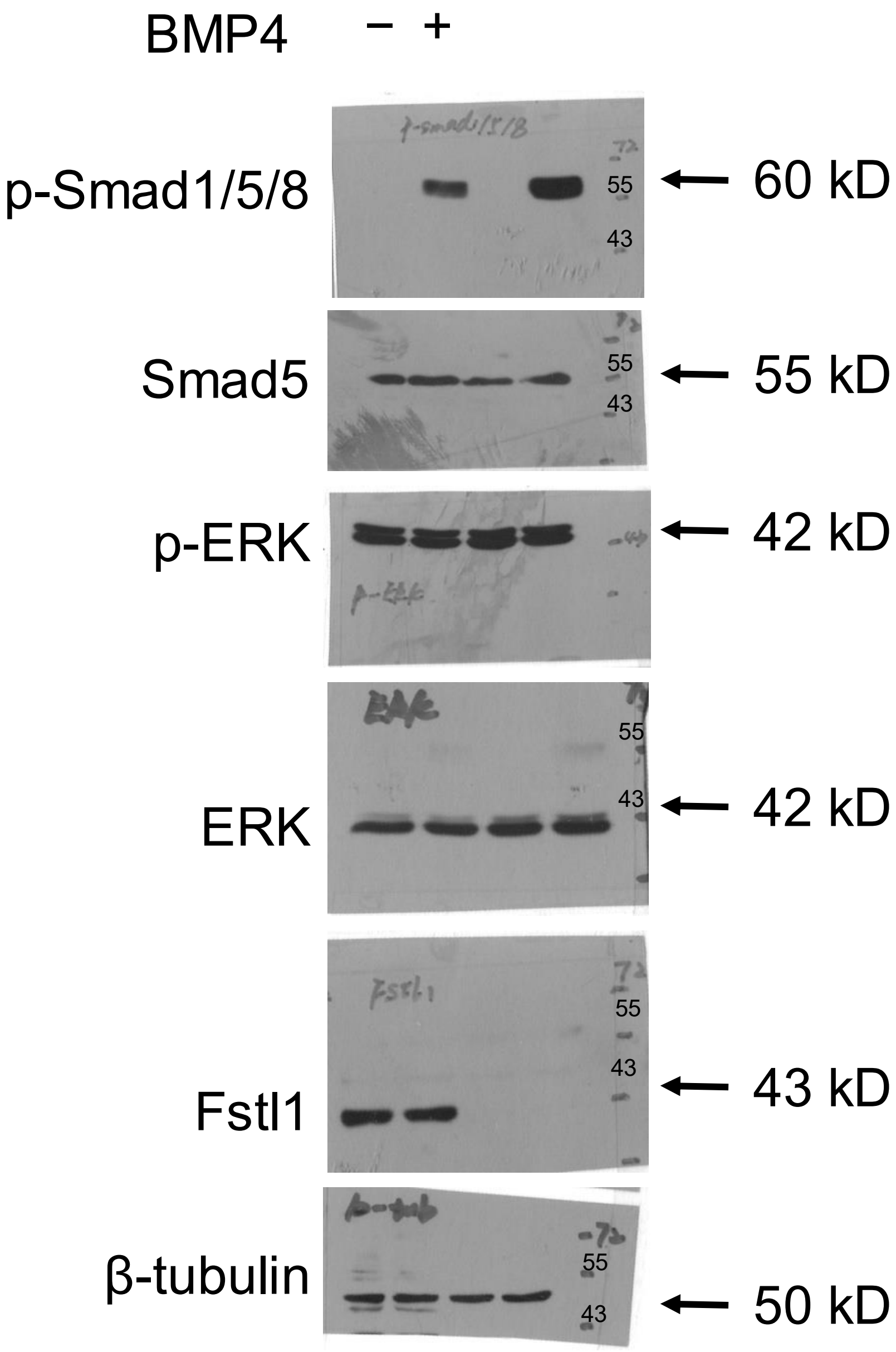

The first independent experiment.

Figure S6A

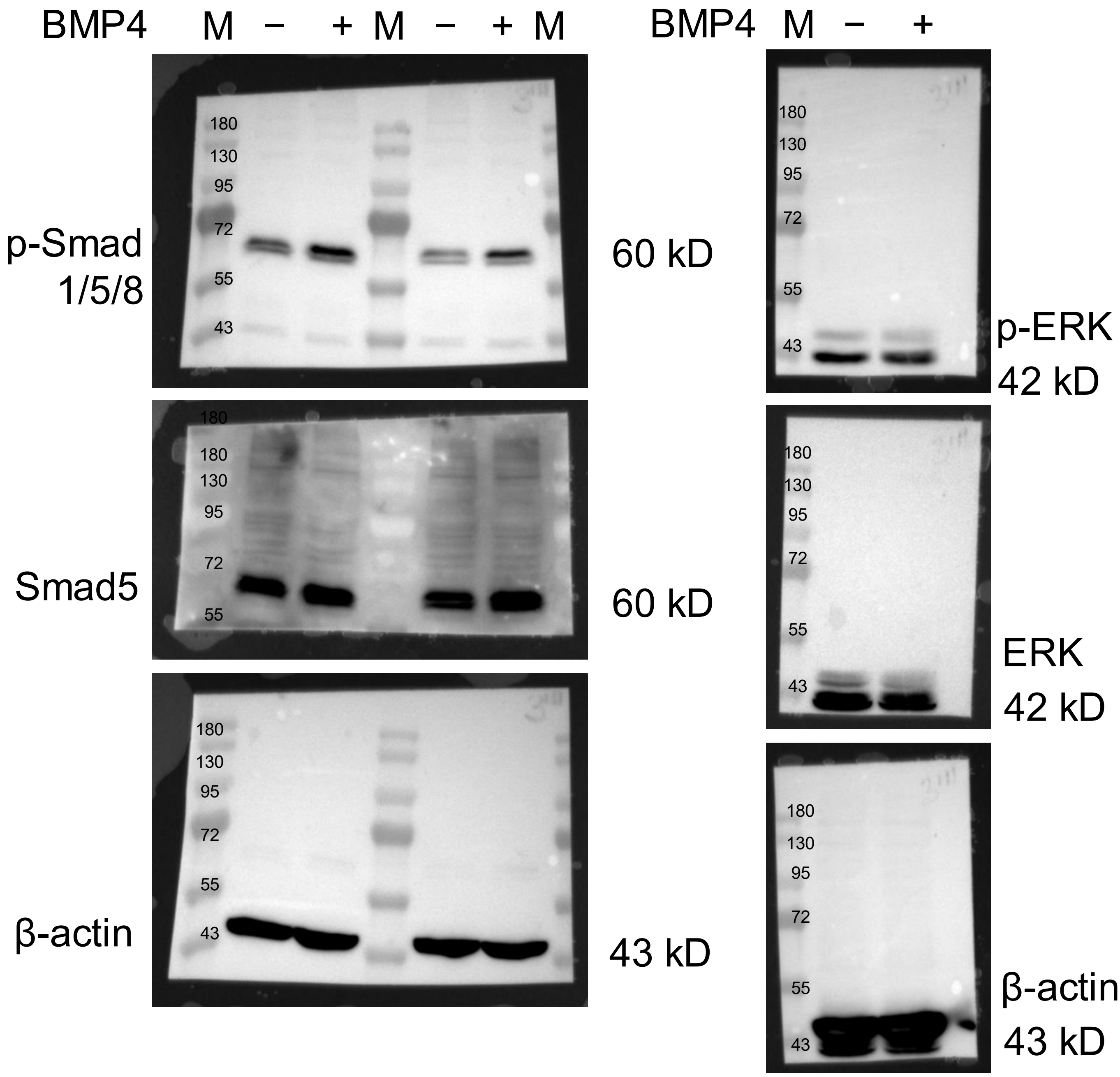

The second independent experiment.

Figure S7C and S7E

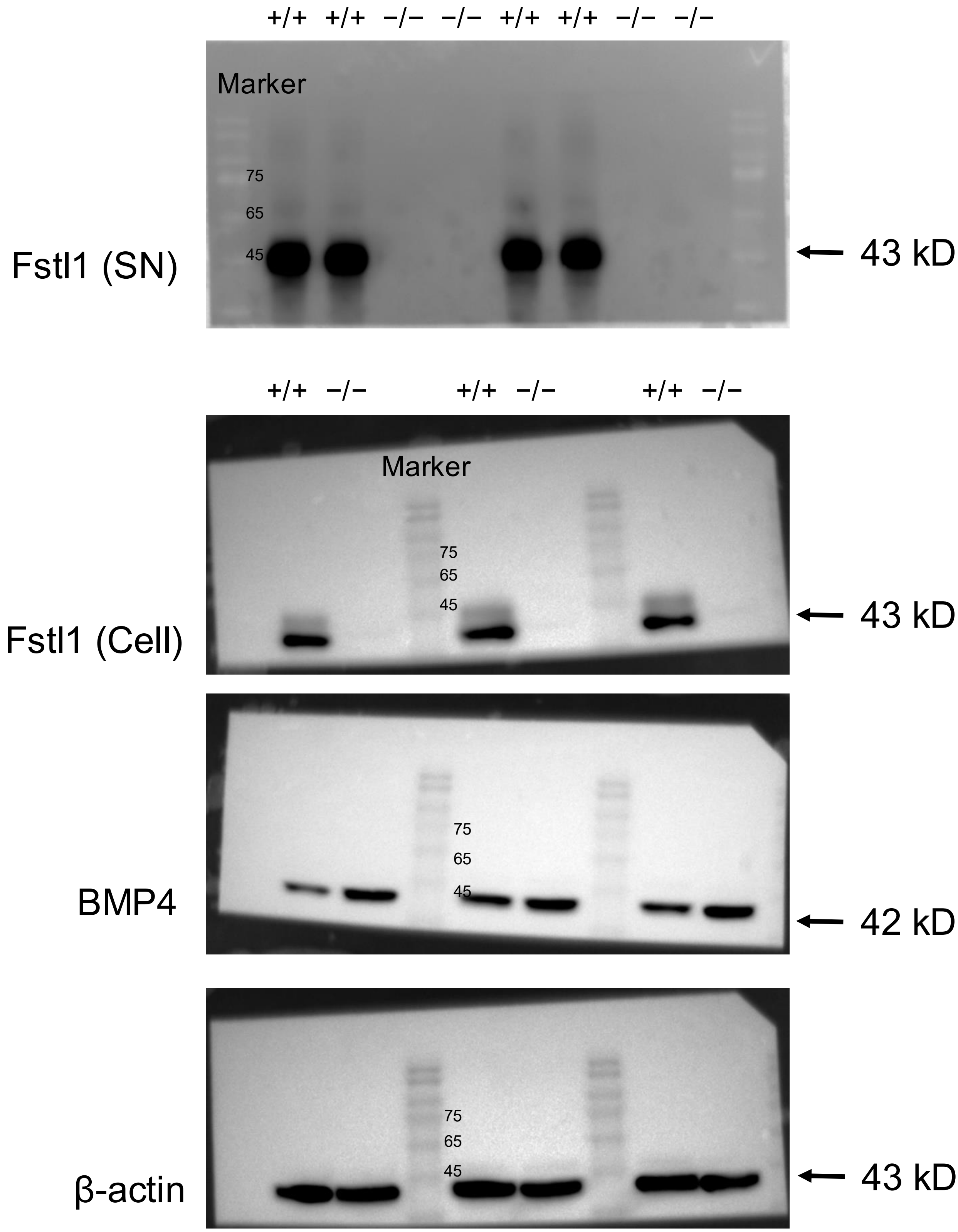

Supplement: Supplementary file 1 [file mmc1.pdf]
